# POWERDRESS-mediated histone deacetylation is essential for thermomorphogenesis in *Arabidopsis thaliana*

**DOI:** 10.1371/journal.pgen.1007280

**Published:** 2018-03-16

**Authors:** Celine Tasset, Avilash Singh Yadav, Sridevi Sureshkumar, Rupali Singh, Lennard van der Woude, Maxim Nekrasov, David Tremethick, Martijn van Zanten, Sureshkumar Balasubramanian

**Affiliations:** 1 School of Biological Sciences, Monash University, Melbourne, VIC, Australia; 2 Molecular Plant Physiology, Institute of Environmental Biology, Utrecht University, Utrecht, The Netherlands; 3 The John Curtin School of Medical Research, Australian National University, Canberra, ACT, Australia; University of Washington, UNITED STATES

## Abstract

Ambient temperature affects plant growth and even minor changes can substantially impact crop yields. The underlying mechanisms of temperature perception and response are just beginning to emerge. Chromatin remodeling, via the eviction of the histone variant H2A.Z containing nucleosomes, is a critical component of thermal response in plants. However, the role of histone modifications remains unknown. Here, through a forward genetic screen, we identify POWERDRESS (PWR), a SANT-domain containing protein known to interact with HISTONE DEACETYLASE 9 (HDA9), as a novel factor required for thermomorphogenesis in *Arabidopsis thaliana*. We show that mutations in *PWR* impede thermomorphogenesis, exemplified by attenuated warm temperature-induced hypocotyl/petiole elongation and early flowering. We show that inhibitors of histone deacetylases diminish temperature-induced hypocotyl elongation, which demonstrates a requirement for histone deacetylation in thermomorphogenesis. We also show that elevated temperature is associated with deacetylation of H3K9 at the +1 nucleosomes of *PHYTOCHROME INTERACTING FACTOR4 (PIF4)* and *YUCCA8 (YUC8)*, and that *PWR* is required for this response. There is global misregulation of genes in *pwr* mutants at elevated temperatures. Meta-analysis revealed that genes that are misregulated in *pwr* mutants display a significant overlap with genes that are H2A.Z-enriched in their gene bodies, and with genes that are differentially expressed in mutants of the components of the SWR1 complex that deposits H2A.Z. Our findings thus uncover a role for *PWR* in facilitating thermomorphogenesis and suggest a potential link between histone deacetylation and H2A.Z nucleosome dynamics in plants.

## Author summary

Plant growth and development is influenced by a variety of external environmental cues. Ambient temperature affects almost all stages of plant development but the underlying molecular mechanisms remain largely unknown. In this paper, the authors show that histone deacetylation, an important chromatin remodeling processes, is essential for eliciting warm temperature-induced growth responses in plants; a process called thermomorphogenesis. The authors identify POWERDRESS, a protein known to interact with HISTONE DEACETYLASE 9, as a novel player essential for thermomorphogenesis in Arabidopsis. Another chromatin remodeling mechanism that is known to play a role in thermal response is the eviction of histone variant H2A.Z containing nucleosomes. Through transcriptome studies and meta-analysis, the authors demonstrate statistical associations between gene regulations conferred through PWR-mediated histone H3 deacetylation and those conferred by histone H2A.Z eviction/incorporation dynamics. This study identifies a novel gene that is essential for thermomorphogenesis and points to a possible link between two seemingly distinct chromatin-remodeling processes in regulating gene expression in plants.

## Introduction

Ambient temperature is one of the major environmental factors that has a significant impact on plant growth and development [1]. Minor changes in temperature can modulate life history traits such as flowering time and seed set [2, 3]. Elevated temperatures modulate plant growth and development in a process termed “thermomorphogenesis” that results in a suite of phenotypes, including an increase in hypocotyl elongation, petiole elongation and early flowering [1]. Thermomorphogenesis is also associated with a dampening of defense responses [4]. The molecular basis of thermal response is just beginning to emerge and involves changes at the level of transcription [1]. Recent work suggests that thermal cues are in part perceived through the phytochrome photoreceptors [5, 6]. For example, phyB has been shown to bind to the promoters of its target genes in a temperature-dependent manner modulating transcriptional response to temperature [5].

One of the other molecular events involved in thermal response is chromatin remodeling. Warm temperatures have been shown to lead to the eviction of the H2A.Z nucleosomes associated with thermo-responsive genes, improving chromatin accessibility resulting in changes in gene expression [7, 8]. H2A.Z nucleosome dynamics appears to be critical not only for temperature responses, but also for general response to external stimuli [9, 10]. H2A.Z deposition is often negatively correlated with gene expression; it is enriched in gene bodies of the “responsive genes”, and *h2a*.*z* mutants display mis-regulation of genes associated with response to the environment [9–11]. The incorporation of H2A.Z on to nucleosomes is mediated through the SWR1 complex in Arabidopsis that consists of proteins encoded by *ACTIN RELATED PROTEIN 6 (ARP6)*, *SWC6* and *PHOTOPERIOD INDEPENDENT EARLY FLOWERING 1 (PIE1)*. Mutations in these genes result in pleiotropic phenotypes [12–15]. In contrast to our understanding of the temperature-induced eviction of H2A.Z-nucleosomes, very little is known about the global role of other chromatin remodeling processes such as histone modifications in ambient temperature response [1, 16].

A central integrator in the transcriptional network regulating thermomorphogenesis is *PHYTOCHROME INTERACTING FACTOR 4 (PIF4)*, which encodes a transcription factor that mediates several temperature-associated phenotypes [1, 17–22]. PIF4 is regulated at multiple levels via complex transcriptional and post-transcriptional regulatory mechanisms in response to temperature [1]. PIF4 subsequently regulates downstream target genes, primarily through transcription [1]. For example, PIF4 induces temperature-induced hypocotyl elongation by stimulating auxin biosynthesis via direct binding to the promoters of auxin biosynthesis genes, including *YUCCA8* [17–19, 23]. Thus, transcriptional responses at multiple levels play critical roles in governing temperature responses in plants.

Here, we identify POWERDRESS (PWR), which is known to interact with HISTONE DEACETYLASE 9 (HDA9) [24, 25], as a novel factor that is essential for thermomorphogenesis in *Arabidopsis*, and uncover a central role for histone deacetylation in mediating thermal responses. We demonstrate that blocking histone deacetylation abolishes temperature-induced hypocotyl elongation. We further show that histone deacetylation is required for the expression of *PIF4* and *YUC8*. Furthermore, we found that elevated temperature is associated with H3K9 deacetylation at the +1 nucleosomes of *PIF4* and *YUC8* and that *PWR* is required for this response. Through RNA-seq experiments, we show that *PWR* suppresses defense gene expression at elevated temperatures. Meta-analysis using of our data and other published datasets revealed a significant overlap between genes that are mis-regulated in *pwr* with those that are modulated through H2A.Z-nucleosome dynamics. Overall, our findings reveal a global role for histone deacetylation in thermal response. In addition, our findings also suggest a statistical association between two distinct chromatin remodeling mechanisms *viz*. histone H3 deacetylation and H2A.Z nucleosome dynamics, in regulating gene expression that extends beyond thermal responses.

## Results

**Fig 1:**
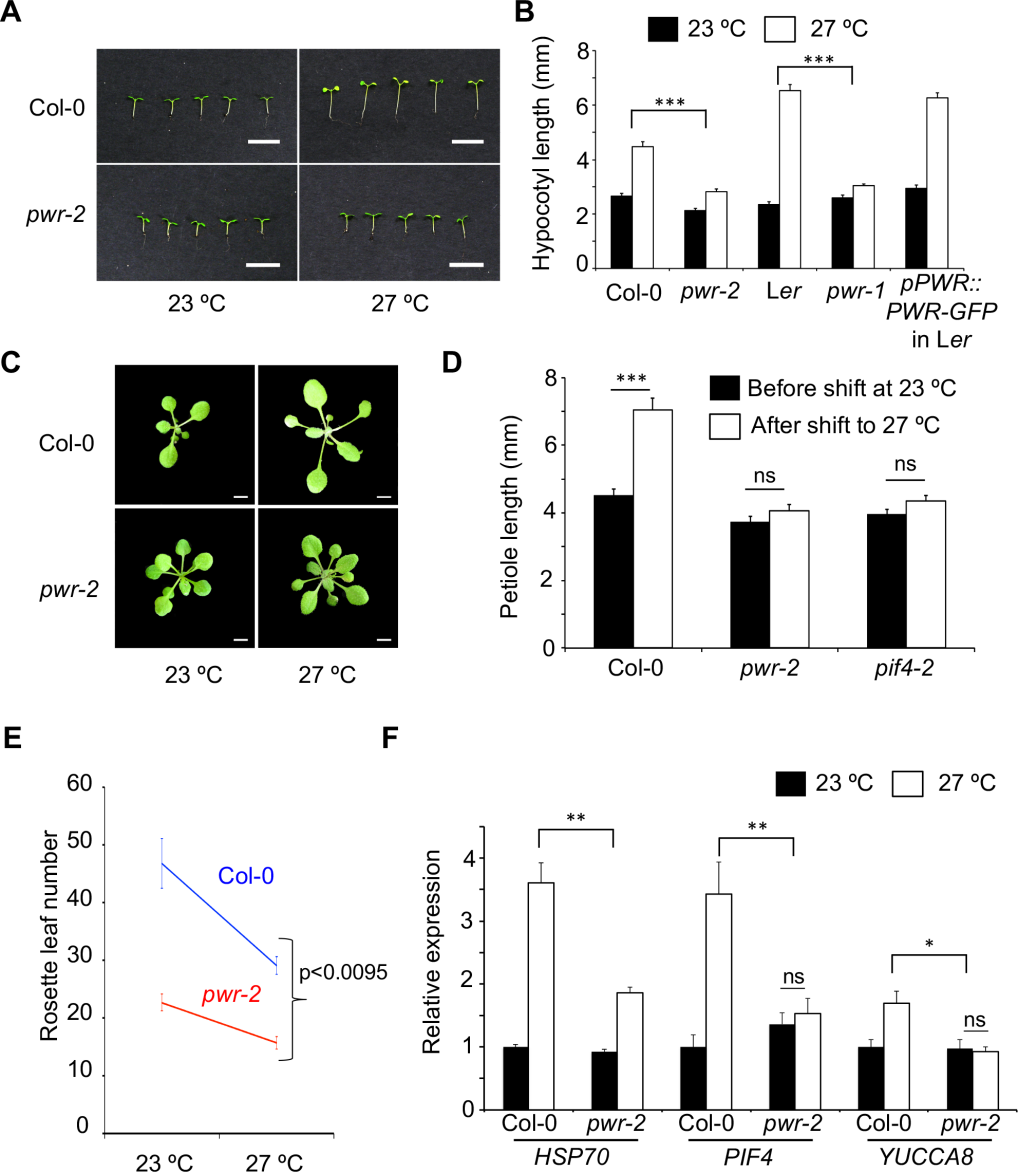
Mutations in *PWR* attenuate thermal responses. A) Hypocotyl lengths of *pwr-2* compared to Col-0 at two different temperatures in short days. B) Hypocotyl length of various genotypes at different temperatures. The *p-value* for the GxE interactions is shown above the bar graphs. *pwr-2* is in the Col-0 background. *pwr-1* and *pPWR*::*PWR-GFP* are in the L*er* background. N = 15 for all samples. C) Petiole elongation of Col-0 and *pwr-2* two days after shift to 27°C. The same plants are shown before and after the shift. D) Quantification of petiole elongation. N = 23–45. E) Flowering time measured as rosette leaf number in Col-0 and *pwr-2* at 23°C and 27°C with *p-valu*e for GxE interaction. F) Relative expression levels of *HSP70*, *PIF4* and *YUCCA8* in Col-0 and *pwr-2*, in 2-week old seedlings at 23°C and 27°C. Data are averages from three independent biological replicates, with each representing approximately 25–30 seedlings. *p-value* for the GxE interaction is shown. *p-values*: ***<0.0001, **<0.001, *<0.05, ns = not significant. Error bars represent standard error. Scale bars: A – 10 mm; C – 5mm.

Elevated temperatures result in increased hypocotyl elongation in *Arabidopsis thaliana* [23]. To identify new genes that facilitate thermomorphogenesis, we carried out a forward genetic screen for temperature-insensitivity in hypocotyl elongation. T-DNA insertion lines [26] allow simultaneous screening of phenotypes at multiple conditions. We screened more than 7000 lines at 23°C and 27°C for attenuated response in temperature-induced hypocotyl elongation and identified 4 potential mutants with altered thermal response. One of the lines that carried an insertion at At3g52250/*POWERDRESS (PWR)* displayed a severely diminished thermal response in hypocotyl elongation (Fig 1A and 1B, *pGxE<0*.*0001*). We next examined additional independent lines having T-DNA insertions at this locus and found that these also display a reduced thermal response, which suggests that *PWR* is essential for temperature-induced hypocotyl elongation (Figs 1B and S1). An *ems-*induced mutant for *PWR (pwr-1)* has been previously isolated as an enhancer of *agamous* [27]. This *pwr-1* allele in the L*er* background also displayed an impairment of temperature-induced hypocotyl elongation (Fig 1B, *pGxE<0*.*0001*), which was complemented in the *pPWR*::*PWR-GFP* line. This independently confirms that *PWR* is the causal locus for the attenuated thermal response (Fig 1B).

To assess whether mutations in *PWR* specifically affects hypocotyl elongation, or generally impairs thermomorphogenesis, we evaluated other temperature-associated phenotypes. Elevated temperatures increase petiole length [17] (S2A Fig) and plants, when shifted from 23°C to 27°C display an elongated petiole within 2 days (Fig 1C and 1D). This marked response to temperature-shift was not observed in *pwr-2* mutants (Fig 1C and 1D). Higher temperatures result in early flowering in Arabidopsis [3]. While mutations in *PWR* also result in early flowering [27] (S2B Fig), the thermo-sensitivity of floral induction was significantly reduced in *pwr-2* (Fig 1E, *pGxE<0*.*0095*). In addition, *pwr-2* mutants appeared smaller than wild type plants (S2B Fig), which suggests that there is a general impairment of plant growth. The observed reduction in temperature-sensitivity correlated with an attenuated response in the temperature-induced expression of *HSP70*, *PIF4* and *YUCCA8* (Fig 1F), genes known to be induced [3, 17, 19] upon elevated temperatures. Taken together these results suggest that *pwr* mutants are generally impaired in thermomorphogenesis.

**Fig 2:**
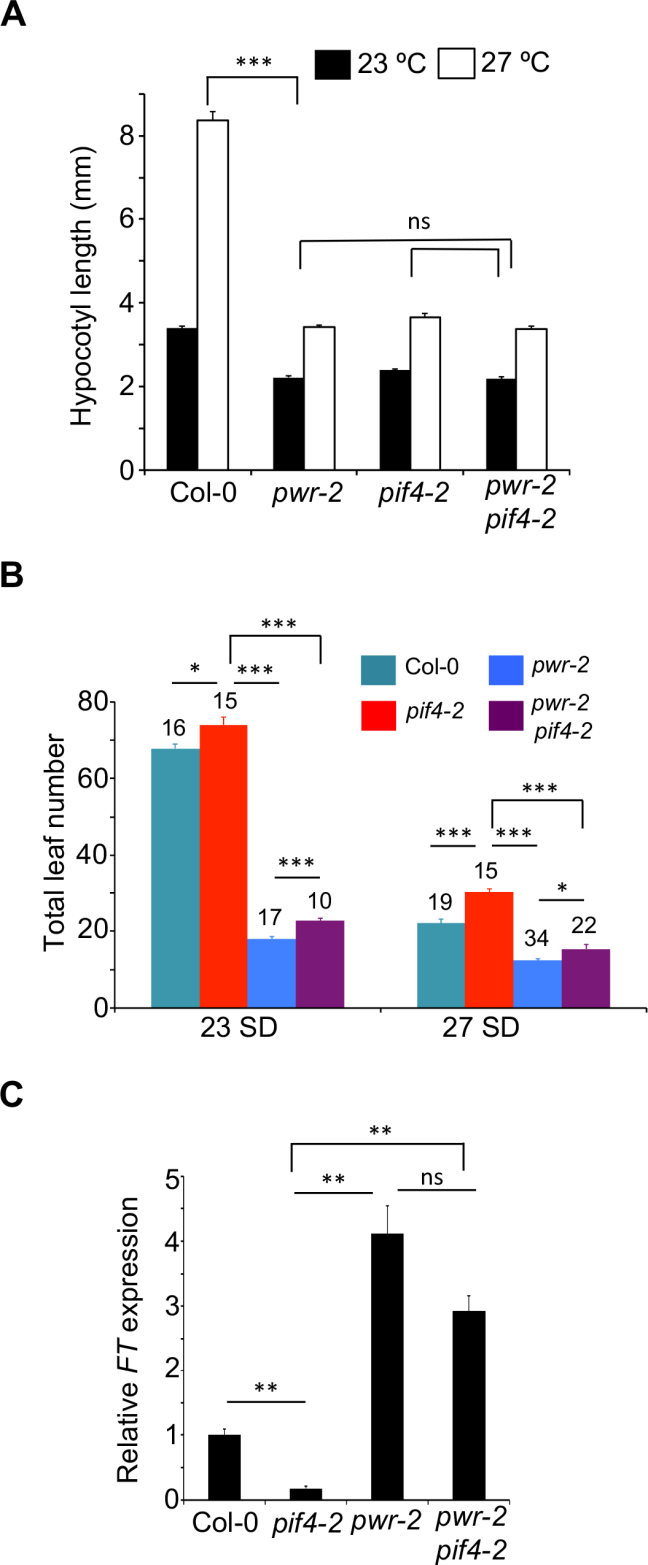
*PWR* acts upstream of *PIF4*. A) Hypocotyl lengths of various genotypes at 23°C and 27°C. *p-values* for the corresponding GxE interactions determined through ANOVA are shown. B) Flowering time measured as total leaf number in *pif4-2 pwr-2* double mutants compared to single mutants at two different temperatures in short days (please see S4 Fig for the data with *pif4-101)*. Number of plants and the *p-values* determined by Student’s t-test are shown above bar graphs. C) Relative *FT* expression levels in different genotypes at 27°C. The data is normalized against the Col-0 reference with *TUBULIN* as internal control. Error bars represent standard error. *p-values*: ***<0.0001, **<0.001, *<0.05, ns = not significant.

The attenuated expression of *PIF4* and *YUCCA8* in *pwr-2* (Fig 1F) suggests that *PWR* is required for temperature-induced auxin biosynthesis, which could be the underlying mechanism for the impaired thermal response in hypocotyl elongation. *PWR* expression remained mostly unaltered in the *pif4-2* mutants (S3 Fig), which suggests that *PIF4* does not regulate *PWR* at the transcriptional level. Consistent with the idea that *PWR* and *PIF4* act in the same genetic cascade, the *pif4 pwr* double mutants were not significantly different from either single mutant, *i*.*e*. no additive or antagonistic interactions were observed in temperature-induced hypocotyl elongation (Figs 2A and S4A). However, *pwr* and *pif4* mutants significantly differ in their flowering phenotype with the *pwr* mutants displaying early flowering at both 23°C and 27°C [27–29] (Fig 1E). We found *pwr-2 pif4-2* and *pwr-2 pif4-101* double mutants to be early flowering similar to *pwr-2* mutants (Figs 2B and S4B). This early flowering at 27°C was associated with an increase in *FT* expression, which suggests that the loss-of-*PWR* can overcome the proposed *PIF4* requirement [28] for *FT* expression at high ambient temperatures (Fig 2C).

**Fig 3:**
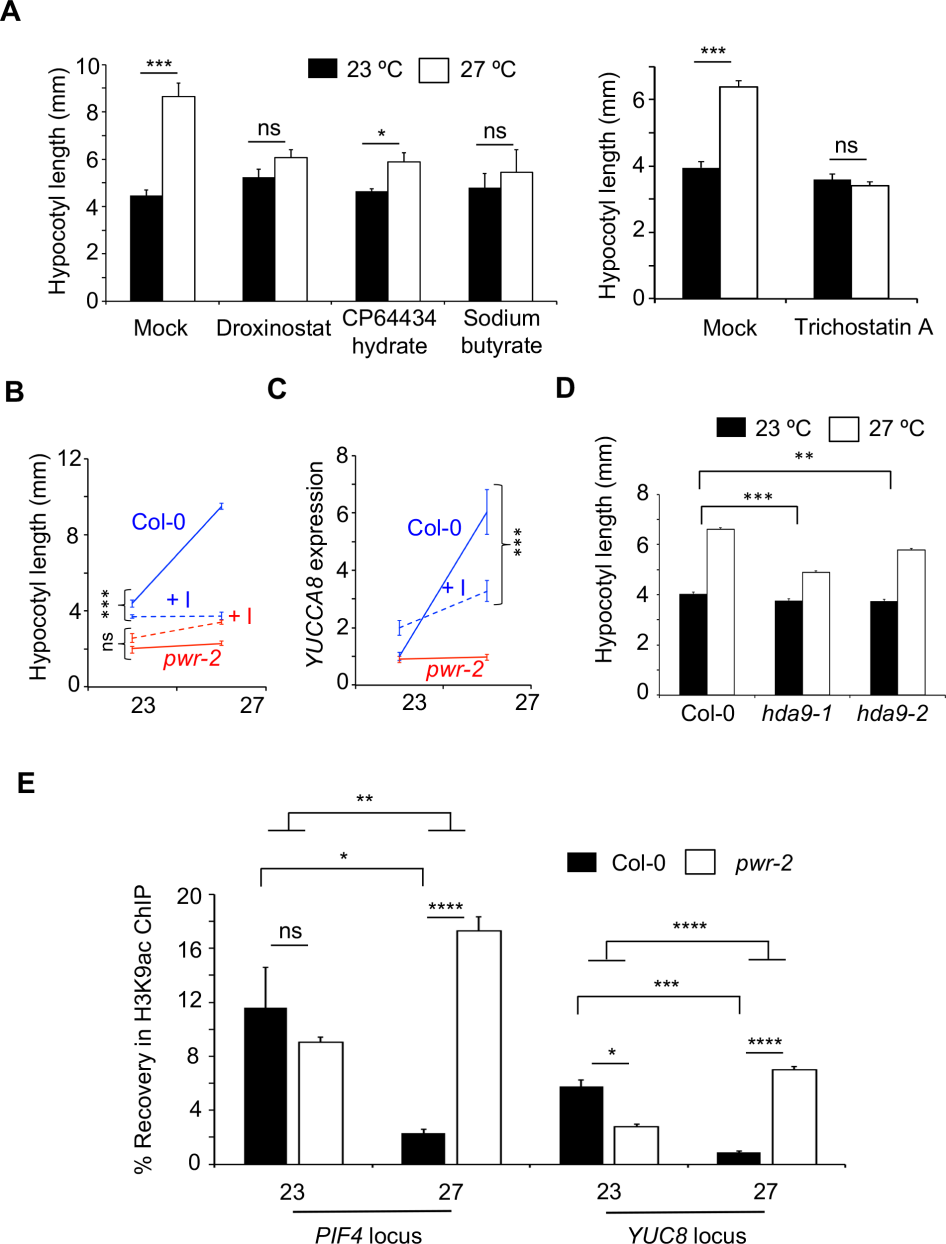
Histone deacetylation is essential for temperature-induced hypocotyl elongation. A) Hypocotyl lengths of Col-0 plants grown in presence of different deacetylase inhibitors (at 10uM except for Trichostatin A (1uM) and a mock control at 23°C and 27°C. *p-values* for differences in hypocotyl elongation between 23°C and 27°C determined by a Student’s t-test N>15. B) Quantitative genetic interaction between genotype and histone deacetylase inhibition. The reaction norms for hypocotyl length in Col-0 (blue) and *pwr-2* (red) are shown in the presence (dashed lines, +I) or absence (solid lines) of Droxinostat. The drug x genotype interaction is shown. C) Reaction norms of the expression levels of *YUCCA8* in Col-0 and *pwr-2*. The drug x genotype interaction for Col-0 is shown. D) Hypocotyl lengths of *hda9* mutants at 22°C or 27°C. N>200. E) Recovery of the DNA fragments spanning the +1 nucleosomes at the *PIF4* and *YUC8* locus after immunoprecipitation of the nuclei with anti-H3K9ac antibodies through ChIP experiments. Error bars indicate standard error. *p-values*: ***<0.0001, **<0.001, *<0.05, ns = not significant.

PWR contains a SANT domain that has been suggested to play role in regulating chromatin accessibility by mediating the interaction between histone tails and the histone modifying enzymes [30]. To assess whether the acetylation status of histones modulate temperature-induced hypocotyl elongation, we grew plants in presence of histone acetylation/deacetylation inhibitors. While we did not detect any difference in hypocotyl length in the presence of histone acetylation inhibitor curcumin (S5 Fig), temperature-induced hypocotyl elongation was severely compromised in plants grown in the presence of different histone deacetylase (HDAC) inhibitors; *viz*. sodium butyrate, Droxinostat, CP64434 hydrate or Trichostatin A (Figs 3A, S6A and S6B). Western blots confirmed inhibition of deacetylation with an increase in acetylated proteins in the presence of sodium butyrate (S6C Fig). These findings confirm that histone deacetylation is essential for thermomorphogenesis. Comparison of the effect of HDAC inhibitors on *pwr-2* mutants, with wild type Col-0, revealed significant drug x temperature (Figs 3B and S6B) and drug x genotype (S6D Fig) interactions confirming that the effect of histone deacetylation depends on the genotype and temperature. The effect of HDAC inhibitors were less pronounced in *pwr-2* compared to Col-0, suggesting that *PWR* acts in the same pathway that is targeted by the HDAC inhibitors. Correlating with the phenotypes, there was a reduction in temperature-induced expression of *YUCCA8* and *PIF4* in presence of HDAC inhibitors (Figs 3C and S6E). Recent studies have shown that PWR physically interacts with HDA9 to target specific loci across the genomes and cause H3K9 deacetylation [24, 25]. Consistent with our findings, we found that *hda9* mutants also displayed attenuated responses in temperature-induced hypocotyl elongation (Fig 3D). Taken together these data suggests that PWR-mediated histone deacetylation is essential for thermomorphogenesis in Arabidopsis.

Elevated expression of *PIF4* and *YUC8* is required for temperature-induced hypocotyl elongation [17–19, 23]. Since temperature-induced hypocotyl elongation also requires PWR-mediated histone deacetylation, we assessed the H3K9 acetylation status of the +1nucleosome of *PIF4* and *YUC8* at different temperatures in Col-0 and *pwr-2* using ChIP experiments. We observed a significant reduction in H3K9acetylation levels at 27°C compared to 23°C in Col-0 (Fig 3E); consistent with our finding (Figs 3C and S6E) that histone deacetylation is required for *PIF4/YUC8* expression at higher temperatures. However, in *pwr-2* mutants, this reduction was not seen and H3K9 hyperacetylation was observed at the +1 nucleosomes of both at *PIF4* and *YUC8* locus at 27°C (Fig 3E). The hyperacetylation of H3K9 in *pwr-2* compared to Col-0 suggests that PWR either directly or indirectly modulates *PIF4* chromatin. Alternatively, the temperature-induced eviction of the +1 nucleosome at *PIF4* and *YUC8* in response to warm temperature may be compromised in *pwr-2* mutants.

**Fig 4:**
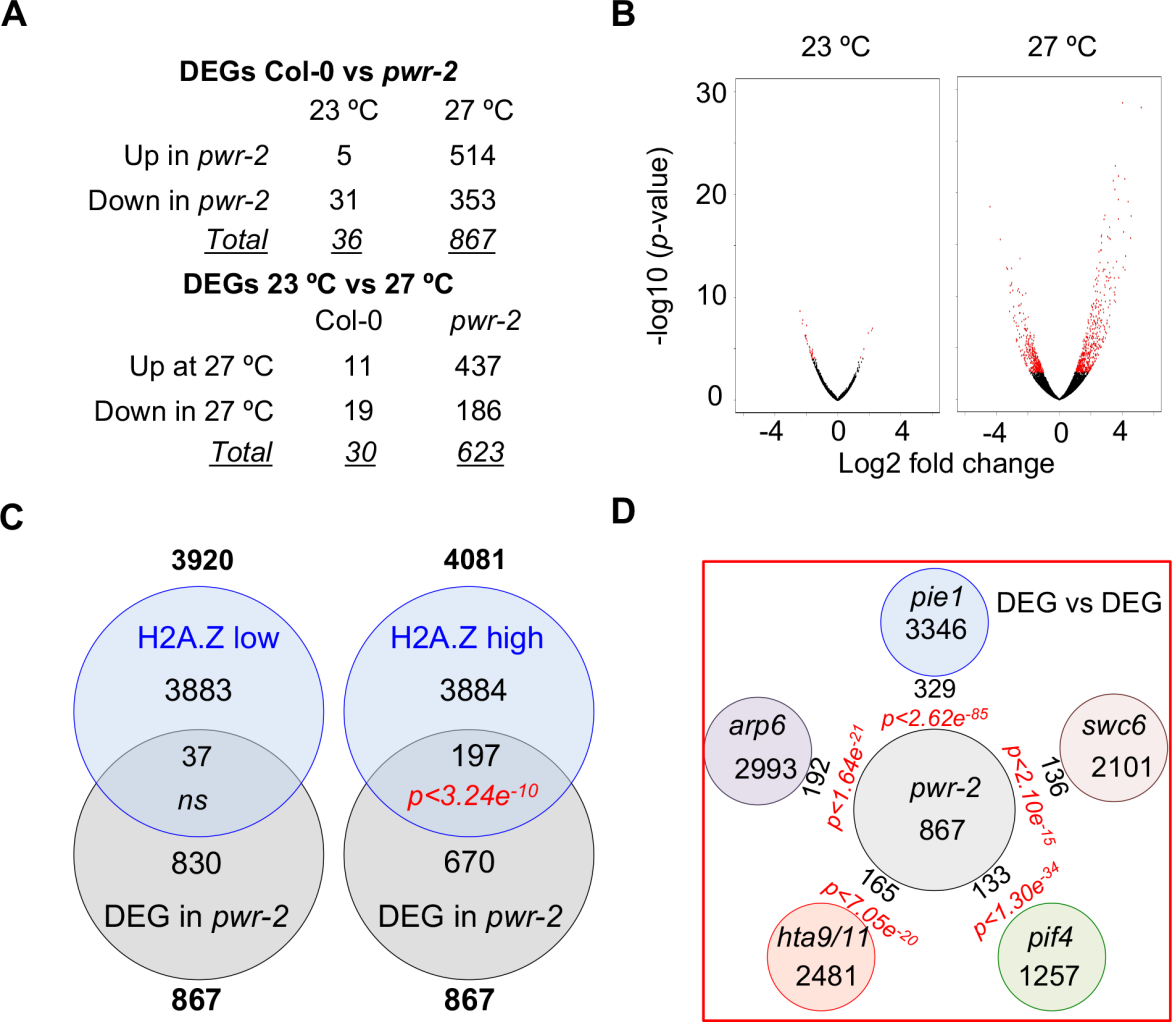
Transcriptome analysis suggests a link between *PWR*-mediated histone deacetylation and H2A.Z nucleosome dynamics. A) Number of DEGs (padj<0.05) between Col-0 and *pwr-2* in seedlings grown at 23°C and/or shifted to 27°C for 2-hours. B) Volcano plots revealing massive mis-regulation of the *pwr-2* transcriptome at high temperatures. Red dots represent DEGs between *pwr-2* and Col-0. C) Overlap of DEGs between *pwr-2* and Col-0 at 27°C with genes that are low-H2A. Z or high-H2A.Z enriched in gene bodies. Total number of DEGs is shown in bold. The H2A.Z data are from [9]. D) Overlap of DEGs between *pwr-2* and Col-0 at 27°C with DEGs in *pie1*, *swc6*, *arp6*, *hta9/hta11* and *pif4*.

Histone deacetylation is typically associated with down regulation of gene expression [31]. The requirement of PWR for thermomorphogenesis therefore indicates that down regulation of gene expression is also critical for a proper thermal response. To obtain further insights into *PWR-*mediated transcriptional regulation in response to temperature, we compared the *pwr-2* and Col-0 transcriptomes at 23°C and 2-hours after a shift to 27°C, in 6-day old seedlings. Interestingly, the number of differentially expressed genes (DEGs) between Col-0 and *pwr-2* was substantially higher at 27°C (867 DEGs), than at 23°C (36 DEGs) (Fig 4A and 4B, S1 and S2 Tables). Analysis of the transcriptomes at 23°C and 27°C identified 30 genes to be differentially expressed in Col-0, while 623 genes were differentially expressed in *pwr-2* (Fig 4A). Thus, the loss of *PWR* resulted in global mis-regulation of transcription at 27°C; this suggests that PWR dampens transcriptional response to elevated temperatures. While the majority of mis-regulated genes were up regulated in *pwr-2* at 27°C, consistent with the role of PWR in histone deacetylation, most of the genes that were induced by higher temperatures in Col-0 (S3 Table) failed to do so in *pwr-2* mutants (S7 Fig).

Gene Ontology (GO) analysis of the DEGs between Col-0 and *pwr-2* at 27°C showed enrichment for GO terms associated with “response” (S4 Table, *Fisher Test with Yekutieli correction*). In addition to enrichment for genes associated with “response to temperature stimuli” *(p<7*.*4e*^*-6*^*)*, various other response terms were also enriched (S4 Table. e.g., response to: chemical stimuli *(p<8*.*7e*^*-59*^*)*, stress *(p<3*.*7e*^*-42*^*)*, carbohydrate *(p<7*.*1e*^*-25*^*)*, other organism *(p<9*.*5e*^*-25*^*)*, water *(p<3*.*4e*^*-21*^*)*, defense *(p<6*.*3e*^*-21*^*)*, hormone *(p<1*.*6e*^*-19*^*)*, ethylene *(p<4*.*1e*^*-12*^*))*. This suggests that PWR, in addition to being involved in temperature response may be generally associated with the transcriptional regulation of “response” genes. A similar GO enrichment profile for “response” was previously reported for genes with high H2A.Z enrichment in gene bodies (here after called high-H2A.Z) [9]. Therefore, we considered whether the genes that are differentially expressed in *pwr-2* overlap with H2A.Z-enriched “response” genes. To assess the significance, we computed the hypergeometric probabilities for the overlap. In addition, we generated 100,000 random pairs of gene lists from Arabidopsis genome, analyzed the overlaps between each pairs of the gene lists, calculated their hypergeometric probabilities and generated a simulated distribution. We then compared where the hypergeometric probabilities for the overlap of analyzed DEGs fell in this distribution (S8 Fig).

We next conducted a meta-analysis of our *pwr* transcriptome data and the published H2A.Z data [9], where genes have been identified with high (high-H2A.Z) or low (low-H2A.Z) H2A.Z enrichment in their gene bodies. A significant overlap was detected between high H2A.Z genes and the DEGs between *pwr-2* and Col-0 (*p<3*.*24e*^*-10*^, *hypergeometric probability test*). In contrast, no significant overlap was found between low-H2A.Z genes and the DEGs between *pwr-2* and Col-0 (Fig 4C). This suggests that the expression of a significant subset of H2A.Z-enriched genes is regulated by PWR.

To assess whether histone acetylation is generally associated with H2A.Z enrichment, we carried out meta-analysis of publicly available data on H3K9acetylation across the genome with H2A.Z enrichment [9, 32]. While H3K9acetylation overlapped with both high-H2A.Z (850/1984, S9A Fig, *p<2e*^*-7*^, *hypergeometric probability test*) and low-H2A.Z (1134/1984, S9A Fig, *p<1*.*71e*^*-60*^, *hypergeometric probability test*) genes, we observed significant overlap of DEGs in *pwr-2* only with H3K9acetylated genes that are also enriched for H2A.Z in their gene bodies (S9B–S9D Fig), which suggests that PWR preferentially modulates high-H2A.Z genes.

Although our findings suggested a statistical association between PWR-mediated histone deacetylation and H2A.Z enrichment, the observed overlap with H2A.Z-enriched genes could be attributed to the thermal transcriptome, as warm temperature also affects H2A.Z nucleosome dynamics [7]. To assess whether there is a general association between H2A.Z enrichment and PWR/HDA9-mediated transcriptional regulation, we carried out a similar meta-analysis with genes that were reported to be differentially expressed in *pwr-2* and *hda9* mutants in published studies, unrelated to temperature but associated with plant aging and flowering [24, 25]. Despite the differences in the sampled tissue, developmental states, growth conditions as well as different research groups being involved, the pattern was similar, where a significant overlap was observed with high-H2A.Z but not with the low-H2A.Z genes (S9 Fig and S10A–S10C Fig). Of the 4081 high H2A.Z genes, we detected 1068 (26%, *p<3*.*79e*^*-29*^, *hypergeometric probability test*) to be differentially expressed in *pwr* and/or *hda9* (S10D Fig). These findings suggest that the statistical association between H2A.Z enrichment and PWR/HDA9 mediated transcriptional regulation not restricted to thermal transcriptome and hints at a potential association between H2A.Z nucleosome dynamics and histone H3 deacetylation.

Histone H3 deacetylation and H2A.Z nucleosome dynamics are two fundamental, yet distinct chromatin-remodeling processes that modulate gene expression in response to diverse environmental stimuli [31, 33]. As our findings suggested a possible previously unexplored potential link between these two, we tested whether changes in gene expression conferred through H2A.Z nucleosome dynamics overlaps with those mediated through histone H3 deacetylation. To assess this, we compared the *pwr-2* and *hda9* mutant transcriptomes with published transcriptomes of H2A.Z mutants *hta9/hta11* (defective for two out of the three H2A.Z encoding genes) [34] and of mutants in components of the SWR1 complex *(arp6 (ACTIN RELATED PROTEIN 6)*, *pie1 (PHOTOPERIOD INDEPENDENT EARLY FLOWERING 1) & swc6)*, which deposits H2A.Z in nucleosomes. In addition, we included the transcriptome for the mutant in *PIF4*, a proposed downstream component of H2A.Z nucleosome dynamics [21, 35]. Here as well, despite the differences in the sampled tissue, developmental states, growth conditions and research groups involved, we observed a significant overlap between the DEGs for all the transcriptomes (Figs 4D and S11–S16), which further supports that there is potential nexus between histone deacetylation and H2A.Z nucleosome dynamics in regulating gene expression.

Among the analyzed transcriptomes, the most significant and discernible overlap was seen with *pie1*, followed by *pif4* (Figs 4D and S11–S17); exhibiting more significance than those of *swc6*, *arp6* or *hta9/hta11* (Figs 4D and S11–S17). *PIE1* also encodes a SANT domain containing protein[13], and its role in the SWR1 complex is well studied [14, 36]. We found *pie1* mutants to display a reduced hypocotyl and petiole elongation response to high temperature, like *pwr-2*, although the effect was less pronounced (S18 Fig). Double mutants of *pwr-2 pie1-6* resembled the *pwr-2* mutants, suggesting that *pwr-2* is epistatic to *pie1* (S18 Fig). Both *PIE1* and *PIF4* play critical roles in plant defense [21, 34, 37]. Defense responses are dampened at higher temperatures [4] and the up regulation of defense response genes in *pwr-2* suggests that *PWR* mediated histone deacetylation could be critical in suppressing defense gene expression at elevated temperatures (S5 Table).

## Discussion

We have demonstrated that *PWR* is required for thermomorphogenesis. Previous studies showed roles for *PWR* in diverse developmental processes including floral determinacy, flowering and senescence [24, 25, 27]. The mechanism through which PWR acts on these processes appears to involve transcriptional regulation by facilitating histone H3 deacetylation. PWR modifies acetylation status through its physical interaction with HDA9 that results in histone deacetylation at specific loci across the genome [24, 25]. Histone deacetylation has been previously shown to be essential in both developmental processes and abiotic stress response [31]. Our results demonstrate that PWR-dependent histone deacetylation is also required for ambient temperature-response in plants.

Chromatin remodeling, through the eviction of H2A.Z-nucleosomes has been previously shown to be critical for thermosensory responses in plants [7]. Our findings on PWR add another layer of chromatin remodeling that is essential in mediating transcriptional responses to temperature. PWR acts at the level of chromatin in conferring thermal response and is thus an upstream factor in the thermosensory response in plants. Our genetic analysis supports this hypothesis. Nevertheless, it remains unclear as to how temperature information is transduced to the PWR/HDA9 complex to regulate these chromatin-remodeling events.

Interestingly, mutations in SWR1 complex components such as *arp6* and *pie1* result in contrasting temperature-induced hypocotyl phenotypes. While *arp6* has long hypocotyls even at lower temperatures [7], mutations in *pie1* result in relatively shorter hypocotyls even at elevated temperatures. The inability of *pie1* mutants to respond to elevated temperature, like *pwr* mutants, reveals the complexity at the level of chromatin remodeling governing thermal responses. It is possible that *PIE1* being a SANT-domain containing protein may have additional roles independent of the SWR1-complex. The striking overlap of *pwr-2* transcriptome with *pie1* when compared to the overlap with *arp6*, *swc6* and *hta9/hta11*, the distinct phenotypes of *pie1* and its genetic interaction with *pwr-2* suggest a broader role of *PIE1;* some of which may be H2A.Z-independent and associated directly or indirectly with the histone deacetylation cascade regulated by PWR.

*PIE1* also plays a critical role in regulating the expression of defense genes and the role of *PIE1* in defense also differs from *ARP6*, *SWC6* and *HTA9/HTA11*[34, 37]. Trade-off between thermosensory growth and defense has recently been suggested to be coordinated by *PIF4*[21]. Our analysis of the up-regulated genes in the *pwr-2* transcriptome also revealed an enrichment of GO terms associated with defense (S5 Table). The strong overlaps of *pwr-2* transcriptome with *pie1* and *pif4* warrants further experiments to test whether PWR-mediated histone H3 deacetylation may also play a role in regulating gene expression changes that are associated with the tradeoff between growth and defense. It is also currently unknown whether *pwr* mutants indeed display enhanced disease resistance, which would be explored in future research. Although *PWR* is required for *PIF4* expression, we do not rule out the possibility that *PWR* may also be required down stream of *PIF4* in regulating defense gene expression.

We have demonstrated that histone deacetylation is an essential aspect of thermomorphogenesis in Arabidopsis and presented data demonstrating a statistical association between gene regulation by histone H3 deacetylation and gene regulation conferred by H2A.Z nucleosome dynamics. This statistical association may hint at a potential link between histone H3 deacetylation and H2A.Z nucleosome dynamics *in planta*. At present it is not clear whether these are independently acting mechanisms or act synergistically in regulating gene expression. Previous studies have suggested that histone H3 acetylation patterns can modulate H2A.Z nucleosome dynamics in yeast [38], whereas H2A.Z has been shown to promote H3 and H4 acetylation in mammalian cells [39, 40]. It is conceivable that these two processes can influence each other and subsequently alter chromatin accessibility resulting in up/down regulation of gene expression. The observed statistical associations are consistent with such a possibility. For example, the absence of *PWR* results in hyperacetylation at the *PIF4* locus; but a consequent reduction in gene expression was observed. Although histone deacetylation is typically associated with suppression of gene expression, there are examples where a need for histone deacetylation for gene expression has been documented. For example, histone deacetylase Hos2 has been shown to be required for gene activity [41, 42]. Loss of Hos2 abolishes cAMP-dependent expression of mating-type genes in *Ustilago maydis* [42]. Our observation that reduction in gene expression associated with hyperacetylation at the *PIF4* locus is also consistent with HDAC inhibitor studies, which show that blocking histone deacetylation results in compromised induction of *PIF4* in response to elevated temperatures. It is conceivable that the eviction of H2A.Z-containing +1nucleosome at *PIF4* locus in response to high temperature may require histone H3 deacetylation. Studies in yeast implicated a role for histone deacetylase complex in H2A.Z nucleosome dynamics [43]. However, clearly further studies would be required to explore this potential link. Exploring the mechanistic basis of this connection at a genome-wide scale would be an exciting avenue for future work.

## Materials and methods

### Plant material and phenotyping

All mutants were in the Col-0 background unless otherwise specified. All T-DNA insertion lines as well as most of the mutant lines used in this study were obtained from the European Arabidopsis Stock Centre. *pwr-1*, *pwr-2*, *hda9-1*, *hda9-2*, *hda6* and *hda19* mutants have been described [27, 44–46]. *pwr-1* and *pPWR::PWR-GFP* lines were gifted by Prof. Xuemei Chen and *pif4-101* is from Prof. Christian Fankhauser. All double mutants were obtained by crossing and confirmed by genotyping. Hypocotyl and petiole length measurements were done as described previously[47]. Briefly, seeds were sterilized, sown on Murashige-Skoog media and then stratified for 2 days at 4°C in dark. The plates were then transferred to CU41L5-Percival growth chambers (Percival Inc, Canada) at 23°C or 27°C in short day conditions (8 hour light / 16 hour dark) and grown vertically for 10 days. For the T-DNA screening, more than 20 seedlings representing each of the 7000 T-DNA lines were grown at 23°C and 27°C and the seedlings were visibly inspected for attenuated response. To quantify the hypocotyl elongation, subsequently, plates with plantlets were imaged and the hypocotyl length was measured using Image J (NIH). All T-DNA lines used in subsequent analysis described in this study were confirmed by using T-DNA insertion using primers listed in the S6 Table. Flowering time measurements were done as described previously and total or rosette leaf number is used as a proxy for flowering time [3]. For the HDAC inhibitor assays all the compounds (Sigma-Aldrich) were dissolved in the described concentration in DMSO and the solvent lacking the compounds was used as a mock control.

### DNA/RNA analyses

DNA and RNA extractions were done as described previously [48]. For gene expression studies DNAse I (Roche)-treated 1ug of total RNA was used for cDNA synthesis using the First strand cDNA synthesis kit (Roche) and the resulting cDNA was diluted and used for realtime PCR analysis with a Lightcycler 480 system (Roche) with SYBR green. The specific primers used for real-time PCR analysis are in S6 Table. Relative expression levels were obtained using the ΔΔcT method [49] using either *UBIQUITIN* or *TUBULIN* as internal controls.

### ChIP experiments and western blots

ChIP experiments were done using a standardized protocol with modifications [50]. Briefly, approximately 1.5g of 10-day old Col-0 and *pwr-2* seedlings grown on plates were collected fixed in formaldehyde. Fixed samples were crushed in liquid nitrogen and the nuclei were isolated from them. The isolated nuclei were subjected to sonication and the sonicated sample was taken for immunoprecipitation. IP was carried out with anti-H3K9ac antibodies (AbCam-ab10812) and the enrichment was analyzed through PCR with the primers described in S6 Table. Western blots were done as described previously [51]. Equal amounts of protein were loaded onto a sodium dodecyl sulfate-polyacrylamide gel electrophoresis (SDS-PAGE), followed by electrophoresis and transfer to Protran BA85 nitrocellulose membranes (Whatman, Germany). Transferred proteins were visualized by Ponceau S red staining. Plant protein samples obtained from *A*. *thaliana* (20 seedlings), were homogenized in 250 μL of Laemmli loading buffer [52].

### Transcriptome studies

RNA-seq analysis was done as described previously [53]. About one hundred 6-day-old seedlings of Col-0 and *pwr*-2 each were grown at 23°C in short days (SD) in growth chambers (GR-36, Percival Scientific, Canada). Half of the samples were moved to 27°C. Tissue from whole seedlings were collected for RNA extraction from both 23°C and 27°C after 2-hours. Two biological replicates were used. Total RNA was extracted from two biological replicates using Isolate II RNA plant kit (Bioline Pty Ltd, Australia). The libraries were prepared and sequenced on an Illumina platform by paired-end sequencing of 90 bp in length at BGI-Shenzen (Beijing Genomics Institute). *FastQC* (http://www.bioinformatics.babraham.ac.uk/projects/fastqc) was used to perform the initial quality control check of the transcriptome data. *SortmeRNA* was used to filter the rRNA sequences from the datasets, using its default rRNA databases comprising of 16S, 18S 23S and 28S rRNAs[54]. The reads for each sample were aligned to *Arabidopsis thaliana* TAIR 10 genome using *Tophat2 (v2*.*1*.*0)* [55] and *bowtie2 (v2*.*1*.*0)* [56]. Raw abundance counts were obtained from the Bio conductor-R-subread package using *featureCounts (v1*.*4*.*5)* [57] from the output produced by *Tophat2*. Only fragments with both reads successfully aligned (specified through–p and–B parameters in *featureCounts*) were considered for summarization. The resulting lists of abundance counts were used as an input data for *DESeq2 (v1*.*14*.*1)* [58] differential expression analysis pipeline. For differential expression analysis and estimation of dispersions across libraries in *DESeq2*, batch effect between replicates was accounted for through a negative binomial GLM as described previously [53]. Genes with a padj<0.05 (Benjamini-Hochberg corrected p-values) were termed as differentially expressed genes (DEGs). The gene lists generated through the analysis of differential expression were used in the online program AgriGO to identify enriched GO terms [59]. Additional gene lists for overlap analysis were either obtained from published data [9, 21, 24, 34, 37]. Overlaps between gene lists were tested through hypergeometric probabilities as well as through simulation studies performed in R. To simulate a distribution, 100,000 random gene lists of comparable sizes to the gene lists that were analyzed (500–2000 genes) were generated from Arabidopsis and the hypergeometric probabilities for the overlaps of such random gene lists were computed. The hypergeometric probabilities for the overlaps observed in the simulated dataset were calculated using hypergeometric probability function in R. The RNA-seq data presented in this paper is available in GEO repository with the accession number GSE101782.

## Supporting information

S1 Fig Identification of other *pwr* alleles. A) T-DNA insertion lines at the *PWR* locus in the Col-0 background, the location of the insert and their corresponding Salk identifiers. Of these *pwr-2* has been previously described [27]. The location of primers used to analyze expression is shown in blue. B) Hypocotyl lengths of different *pwr* alleles at 23°C and 27°C short days. The *p-values* for G x E between the different *pwr* alleles and Col-0 is shown. C) Relative expression levels of *PWR* different mutant alleles grown 23°C and 27°C short days. The *pwr-5* and *pwr-6* alleles have higher *PWR* expression and thus are not RNA-null alleles. The *p-values* for the difference in *PWR* expression between *pwr* alleles and Col-0 at 23°C determined through a Student’s t-test is shown. No significant differences were observed between 23°C and 27°C. Error bars indicate standard error. *p- values*: ***<0.0001, **<0.001, *<0.05, ns = not significant.
(TIF)

S2 Fig Growth differences between Col-0 and *pwr-2* at 27°C. A) Petiole length of Col-0 and *pwr-2* at 23°C and 27°C. The number of petioles measured is shown above the bars. The *p-values* for the difference in petiole lengths between temperature determined through Student’s t-test is shown above the bars. The *p-value* for G x E interaction is also shown at the top. B) *4*-week old Col-0 and *pwr-2* plants grown at 27°C in short days. Note the compact stature and early flowering in *pwr-2* compared to Col-0. Error bars indicate standard error. *p- values*: ***<0.0001, **<0.001, *<0.05, ns = not significant.
(TIF)

S3 Fig *PWR* expression is unaffected in *pif4-2* mutants. Relative expression levels of *PWR* in Col-0 (black) and *pif4-2* (white) at different temperatures (23°C and 27°C) in long (LD) and short (SD) days are shown. Expression is normalized against the *PWR* expression levels in Col-0 for each condition and *TUBULIN* was used as an internal control. *PWR* expression levels in *pwr-2* (grey) are shown as a negative control. Error bars indicate standard error. *p- values*: ***<0.0001, **<0.001, *<0.05, ns = not significant.
(TIF)

S4 Fig Analysis of the genetic interactions between *pwr-2* and *pif4-101*. A) Hypocotyl lengths of various genotypes at 23°C and 27°C. *p-values* for the corresponding GxE interactions determined through ANOVA are shown. B) Flowering time of *pif4-2 pwr-101* double mutants compared to single mutants at two different temperatures. Number of plants and the *P-values* determined through Student’s t-test are shown above bar graphs. Error bars indicate standard error. *p- values*: ***<0.0001, **<0.001, *<0.05, ns = not significant.
(TIF)

S5 Fig Inhibition of histone acetylation does not affect thermomorphogenesis. Hypocotyl elongation in Col-0 and *pwr-2* mutants observed in plants grown in presence of 10uM of curcumin, an inhibitor or histone acetyl transferase compared to mock at 23°C and 27°C.
(TIF)

S6 Fig Histone deacetylation is essential for thermomorphogenesis. A & B) Dose-response effect of Trichostatin-A (A, N = 9 replicates with >20 plants), and Sodium Butyrate (B, N = 8), on Col-0 (A, B) and *pwr-2* (B) at 23°C and 27°C. *p-values* shown are derived from one way ANOVA using the presence/absence of the drug as a factor. C) Western blots of crude plant extracts probed with anti-acetylated antibody from plants grown with or without Sodium Butyrate. Ponceau-S stained gel is shown as loading control. D) Reaction norms of hypocotyl lengths in Mock vs HDAC inhibitor treatment. The *p-value* for the drug x genotype interaction, determined through ANOVA is shown. E) Effect of inhibition of histone deacetylation on *PIF4* expression in seedlings grown at 23°C or 27°C with or without Sodium Butyrate at different concentrations. *p-values* are determined by one way ANOVA with temperature as a factor. Error bars represent standard error. *p- values*: ***<0.0001, **<0.001, *<0.05, ns = not significant.
(TIF)

S7 Fig Genes up regulated in Col-0 at higher temperature are mostly attenuated in *pwr-2*. Comparison of the fold change in expression levels for the 11 genes that are up regulated at 27°C in Col-0 (blue) and their corresponding response in *pwr-2* (red).
(TIF)

S8 Fig Distribution of hypergeometric probabilities obtained from 100,000 simulations of gene overlap analysis by sampling Arabidopsis genome. The top panel represents the frequency distribution of hypergeometric probabilities obtained through simulations. Random gene lists of 500 to 2000 genes were generated by sampling the Arabidopsis genome and the hypergeometric probability was estimated for the gene overlap. The entire analysis is repeated 100,000 times and the distribution of the probabilities is shown. To demonstrate the significance of the overlaps that are shown in this paper, the p-value distribution for those tests that yielded p<0.05 is shown in the second panel (expanded orange box). The p-values have been log-transformed to show the magnitude of the differences. Please note the differences in the scale for X-axis. The p-values for the gene overlaps for *pwr-2* and *hda9* from different labs is shown by green and black arrows respectively. The red-arrows depict the p-values for the gene overlap with DEGs in *pwr-2* from our group and the blue arrows refer to the same for the overlap between the combined dataset for *pwr-2* and other genes. Note all the p-values fall completely outside the distributions obtained through simulations.
(TIF)

S9 Fig Overlap between H2A.Z enrichment, H3K9acetylation and PWR-dependent gene regulation. A) Overlap between genes with H3K9 acetylation and genes with either high or low H2A.Z in their gene bodies B & C) Overlap among genes that are up/down regulated in *pwr-2* with H3K9acetylated genes with high (B) or low (C) H2A.Z. The DEGs data is the union of Tasset et al (current study) and Kim et al [25] study from seedlings. D & E) Overlap among genes that are up/down regulated in *hda9* with H3K9acetylated genes with high (B) or low (C) H2A.Z. The DEGs data is from Kim et al [25]. The significant p-values shown in red represent hypergeometric probability for the overlap. ns = not significant.
(TIF)

S10 Fig Overlap analysis of DEGs with genes that are low-H2A. Z and high-H2A.Z genes. A) Overlap of the DEGs in *hta9/hta11* double mutants with low-H2A.Z and high-H2A.Z genes. B) Overlap of DEGs in *pwr-2* mutants from two different data sets with low-H2A.Z and high-H2A.Z genes are shown. C) Overlap of DEGs in *hda9-1* mutants from two different data sets with low-H2A.Z and high H2A.Z genes are shown. D) Overlap of DEGs in *pwr-2* and/or *hda9* with low-H2A.Z and high-H2A.Z genes are shown. Total numbers of genes in respective gene lists are shown in bold. The data source is shown on top. *p-values* refer to hypergeometric probabilities and the significant *p-values* are shown in red. ns = not significant.
(TIF)

S11 Fig H2A.Z-related transcriptional response overlaps with that of *PWR*. A-E) Overlap of DEGs in *pwr-2* compared to Col-0 at 27°C with DEGs in *pie1*, *swc6*, *pif4*, *hta9/hta11* and *arp6*. A) Overlap among DEGs. B) Overlap among genes that are up regulated in all genotypes. C) Overlap among genes that are down regulated in all genotypes. D) Overlap among genes that were up regulated in *pwr-2*, but down-regulated in other genotypes. E) Overlap among genes that were down-regulated in *pwr-2*, but up regulated in other genotypes. The total number of DEGs is shown in circles and the numbers in between represent the overlapping set of genes. The significant p-values shown in red represent hypergeometric probability for the overlap. ns = not significant. The transcriptome data is from[21, 34, 37].
(TIF)

S12 Fig Overlap analysis of DEGs in *pwr-2* with DEGs in the transcriptomes of *pie1*, *swc6*, *arp6*, *pif4-2* and the *hta9/hta11* double mutants based on Chen et al data. A-E) Overlap of DEGs in *pwr-2* compared to Col-0 with DEGs in *pie1*, *swc6*, *pif4*, *hta9/hta11* and *arp6*. A) Overlap among DEGs. B) Overlap among genes that are up regulated in all genotypes. C) Overlap among genes that are down regulated in all genotypes. D) Overlap among genes that were up regulated in *pwr-2*, but down regulated in other genotypes. E) Overlap among genes that were down regulated in *pwr-2*, but up regulated in other genotypes. The total number of DEGs is shown in circles and the numbers in between represent the overlapping set of genes. The significant p-values shown in red represent hypergeometric probability for the overlap. ns = not significant. The transcriptome data is from [21, 34, 37].
(TIF)

S13 Fig Overlap analysis of DEGs in *pwr-2* with DEGs in the transcriptomes of *pie1*, *swc6*, *arp6*, *pif4-2* and the *hta9/hta11* double mutants based on Kim et al data. A-E) Overlap of DEGs in *pwr-2* compared to Col-0 with DEGs in *pie1*, *swc6*, *pif4*, *hta9/hta11* and *arp6*. A) Overlap among DEGs. B) Overlap among genes that are up regulated in all genotypes. C) Overlap among genes that are down regulated in all genotypes. D) Overlap among genes that were up regulated in *pwr-2*, but down regulated in other genotypes. E) Overlap among genes that were down regulated in *pwr-2*, but up regulated in other genotypes. The total number of DEGs is shown in circles and the numbers in between represent the overlapping set of genes. The significant p-values shown in red represent hypergeometric probability for the overlap. ns = not significant. The transcriptome data is from [21, 34, 37].
(TIF)

S14 Fig Comparison of the overlaps between the transcriptional response observed in *pwr-2* in three different datasets and their overlap with the DEGs in *arp6*, *pie1*, *swc6*, *hta9/hta11* and *pif4*. A) Overlap of DEGs in *pwr* compared to Col-0 compiled from all three datasets (excluding genes that did not change in the same direction in the datasets) with DEGs in *pie1*, *swc6*, *pif4*, *hta9/hta11* and *arp6*. The total number of DEGs is shown in circles and the numbers in between represent the overlapping set of genes. The transcriptome data is from [21, 34, 37]. B) Overlap of the DEGs in *pwr* in the three different datasets. The transcriptome data is from [24, 25] The p-values are shown next to each of the overlaps. C) Overlap among up-regulated genes. D) Overlap among down regulated genes. The total number of DEGs is shown in circles and the numbers in between represent the overlapping set of genes (B-D). The significant *p-values* shown in red represent hypergeometric probability for the overlap. ns = not significant.
(TIF)

S15 Fig Overlap analysis of DEGs in *hda9* with DEGs in the transcriptomes of *pie1*, *swc6*, *arp6*, *pif4-2* and the *hta9/hta11* double mutants based on Chen et al data. A-E) Overlap of DEGs in *hda9* compared to Col-0 with DEGs in *pie1*, *swc6*, *pif4*, *hta9/hta11* and *arp6*. A) Overlap among DEGs. B) Overlap among genes that are up regulated in all genotypes. C) Overlap among genes that are down regulated in all genotypes. D) Overlap among genes that were up regulated in *hda9*, but down regulated in other genotypes. E) Overlap among genes that were down regulated in *hda9*, but up regulated in other genotypes. The total number of DEGs is shown in circles and the numbers in between represent the overlapping set of genes. The significant p-values shown in red represent hypergeometric probability for the overlap. ns = not significant. The transcriptome data is from[21, 34, 37].
(TIF)

S16 Fig Overlap analysis of DEGs in *hda9* with DEGs in the transcriptomes of *pie1*, *swc6*, *arp6*, *pif4-2* and the *hta9/hta11* double mutants based on Kim et al data. A-E) Overlap of DEGs in *hda9* compared to Col-0 in the Kim et al data set with DEGs in *pie1*, *swc6*, *pif4*, *hta9/hta11* and *arp6*. A) Overlap among DEGs. B) Overlap among genes that are up regulated in all genotypes. C) Overlap among genes that are down regulated in all genotypes. D) Overlap among genes that were up regulated in *hda9*, but down regulated in other genotypes. E) Overlap among genes that were down regulated in *hda9*, but up regulated in other genotypes. The total number of DEGs is shown in circles and the numbers in between represent the overlapping set of genes. The significant p-values shown in red represent hypergeometric probability for the overlap. ns = not significant. The transcriptome data is from[21, 24, 25, 34, 37].
(TIF)

S17 Fig Comparison of the overlaps between the transcriptional response observed in *hda9* two different datasets and their overlap with the DEGs in *arp6*, *pie1*, *swc6*, *hta9/hta11* and *pif4*. A) Overlap of DEGs in *hda9* compared to Col-0 compiled from two datasets (excluding genes that did not change in the same direction in the datasets) with DEGs in *pie1*, *swc6*, *pif4*, *hta9/hta11* and *arp6*. The total number of DEGs is shown in circles and the numbers in between represent the overlapping set of genes. The transcriptome data is from [21, 24, 25, 34, 37]. B) Overlap of the DEGs in *pwr* in the three different datasets. The p-values are shown next to each of the overlaps. C) Overlap among up-regulated genes. D) Overlap among down regulated genes. The significant p-values shown in red represent hypergeometric probability for the overlap. ns = not significant.
(TIF)

S18 Fig Mutations in *PIE1* compromise temperature-induced hypocotyl and petiole elongation. A) Hypocotyl lengths of various genotypes at 23°C and 27°C. *P-values* for the corresponding GxE interactions determined through ANOVA are shown. The Col-0 and *pwr-2* data is the same as shown in Fig 2A. B) Comparison of the Col-0 and *pie1* mutant leaves grown at 23°C and 27°C. Scale bar: 1cm. Error bars indicate standard error. *p- values*: ***<0.0001, **<0.001, *<0.05.
(TIF)

S1 Table List of DEGs between Col-0 and *pwr-2* at 23°C. (XLSX)

S2 Table List of DEGs between Col-0 and *pwr-2* at 27°C. (XLSX)

S3 Table List of DEGs between 23°C and 27°C in Col-0. (XLSX)

S4 Table GO-terms that were significantly enriched in DEGs in *pwr-2* mutants. The GO terms with “response” are given in bold.
(XLSX)

S5 Table GO-terms that were significantly enriched in genes that were up-regulated in *pwr-2* mutants. The GO terms with “response” are given in bold. The GO terms associated with “defense” are highlighted.
(XLSX)

S6 Table List of primers in used in this study. (XLSX)
