# Supplementary figures and images for: POWERDRESS-mediated histone deacetylation is essential for thermomorphogenesis in Arabidopsis thaliana
Source: PLoS Genet. 2018 Mar 16;14(3):e1007280. doi: 10.1371/journal.pgen.1007280 (PMC5874081; doi:10.1371/journal.pgen.1007280)

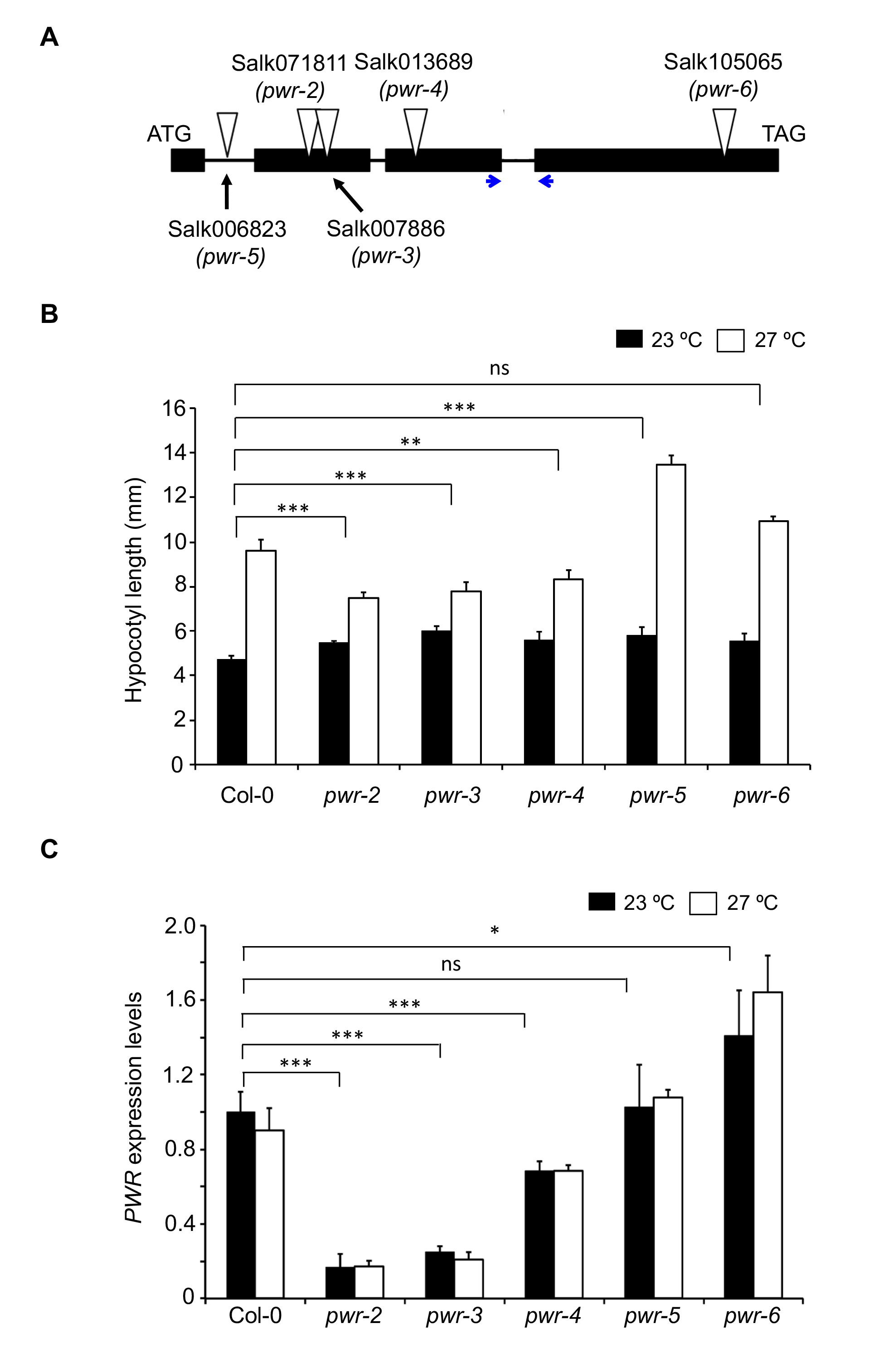

Supplement: S1 Fig — A) T-DNA insertion lines at the PWR locus in the Col-0 background, the location of the insert and their corresponding Salk identifiers. Of these pwr-2 has been previously described [27]. The location of primers used to analyze expression is shown in blue. B) Hypocotyl lengths of different pwr alleles at 23°C and 27°C short days. The p-values for G x E between the different pwr alleles and Col-0 is shown. C) Relative expression levels of PWR different mutant alleles grown 23°C and 27°C short days. The pwr-5 and pwr-6 alleles have higher PWR expression and thus are not RNA-null alleles. The p-values for the difference in PWR expression between pwr alleles and Col-0 at 23°C determined through a Student’s t-test is shown. No significant differences were observed between 23°C and 27°C. Error bars indicate standard error. p- values: ***<0.0001, **<0.001, *<0.05, ns = not significant. (TIF) [file pgen.1007280.s001.tif]

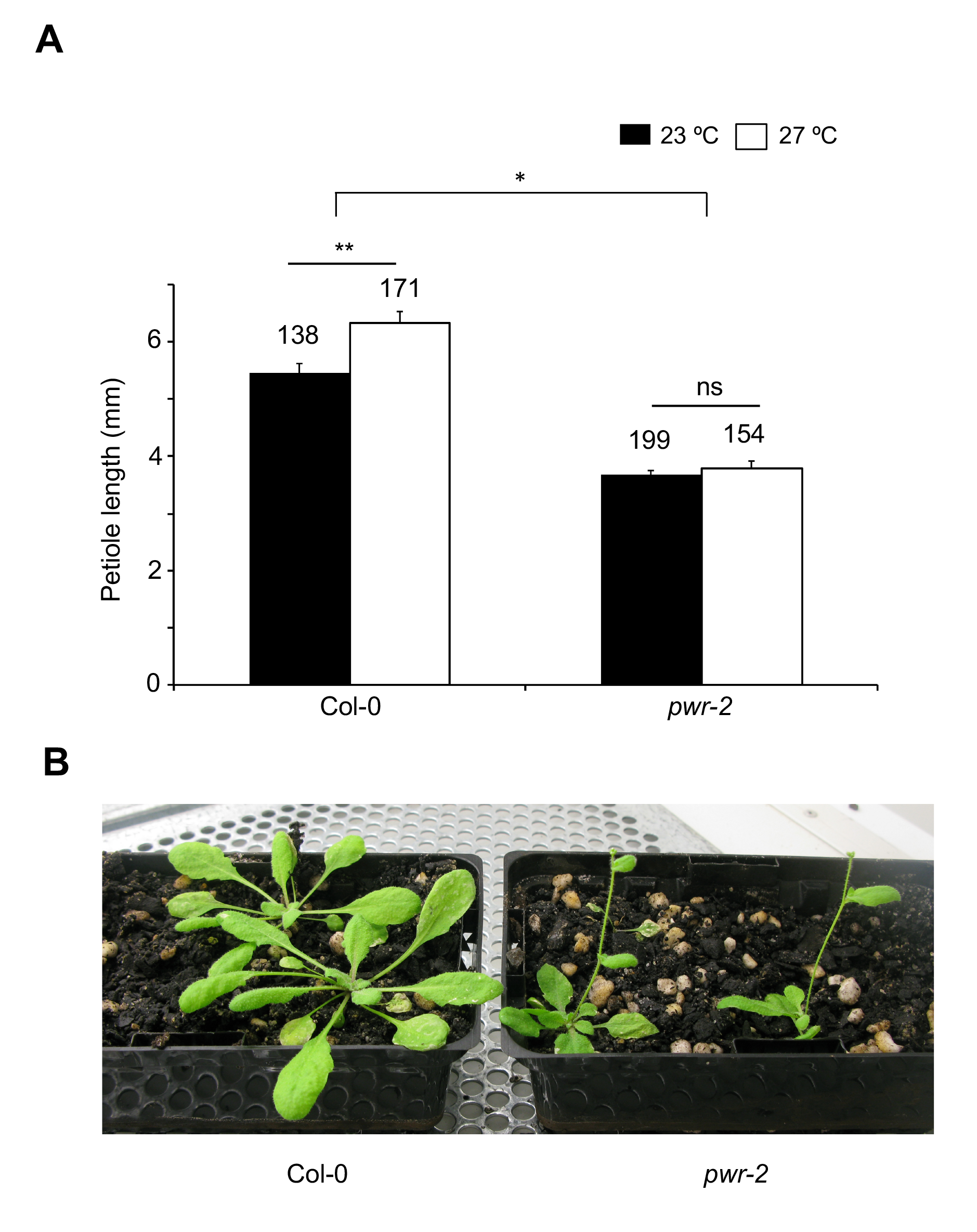

Supplement: S2 Fig — A) Petiole length of Col-0 and pwr-2 at 23°C and 27°C. The number of petioles measured is shown above the bars. The p-values for the difference in petiole lengths between temperature determined through Student’s t-test is shown above the bars. The p-value for G x E interaction is also shown at the top. B) 4-week old Col-0 and pwr-2 plants grown at 27°C in short days. Note the compact stature and early flowering in pwr-2 compared to Col-0. Error bars indicate standard error. p- values: ***<0.0001, **<0.001, *<0.05, ns = not significant. (TIF) [file pgen.1007280.s002.tif]

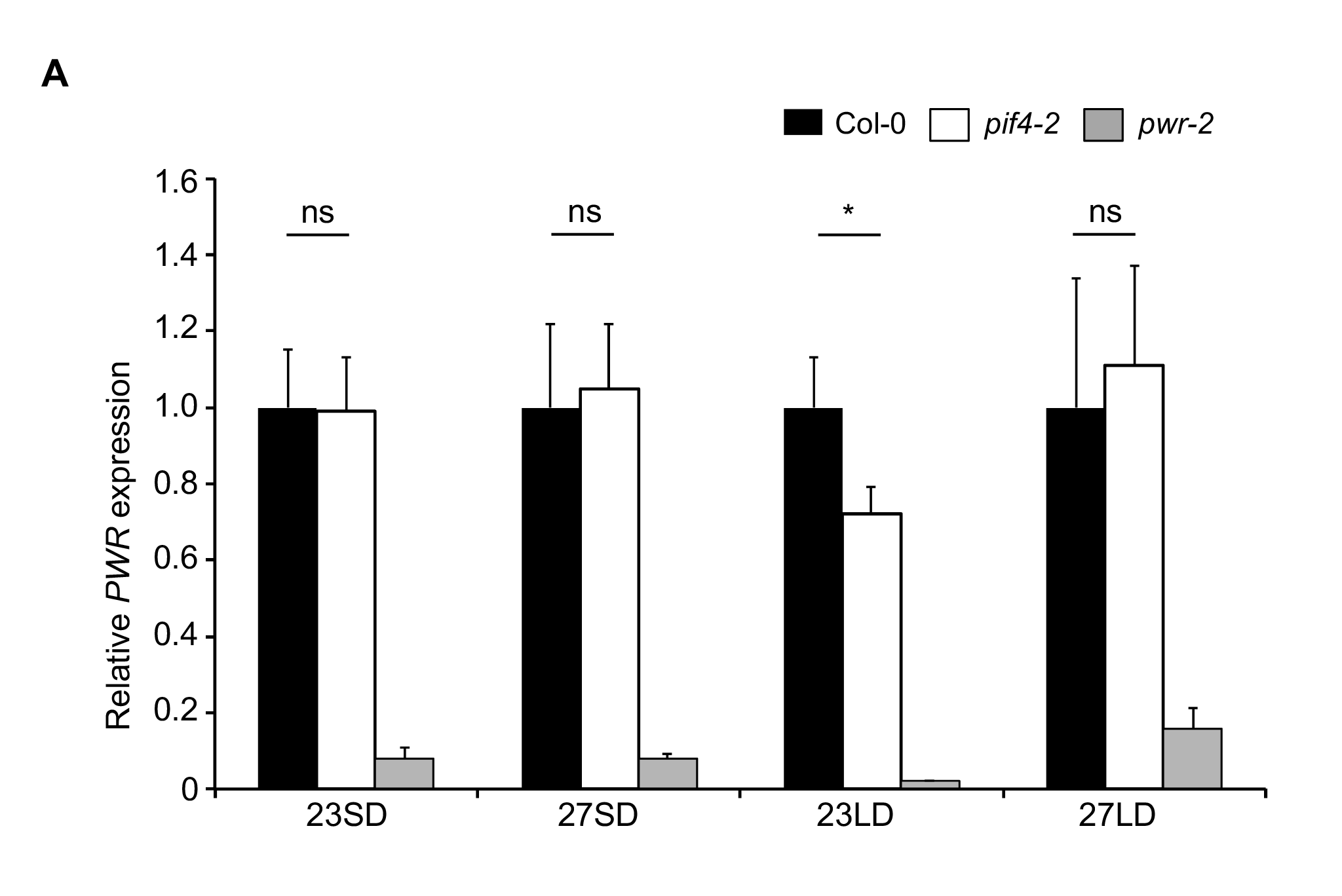

Supplement: S3 Fig — Relative expression levels of PWR in Col-0 (black) and pif4-2 (white) at different temperatures (23°C and 27°C) in long (LD) and short (SD) days are shown. Expression is normalized against the PWR expression levels in Col-0 for each condition and TUBULIN was used as an internal control. PWR expression levels in pwr-2 (grey) are shown as a negative control. Error bars indicate standard error. p- values: ***<0.0001, **<0.001, *<0.05, ns = not significant. (TIF) [file pgen.1007280.s003.tif]

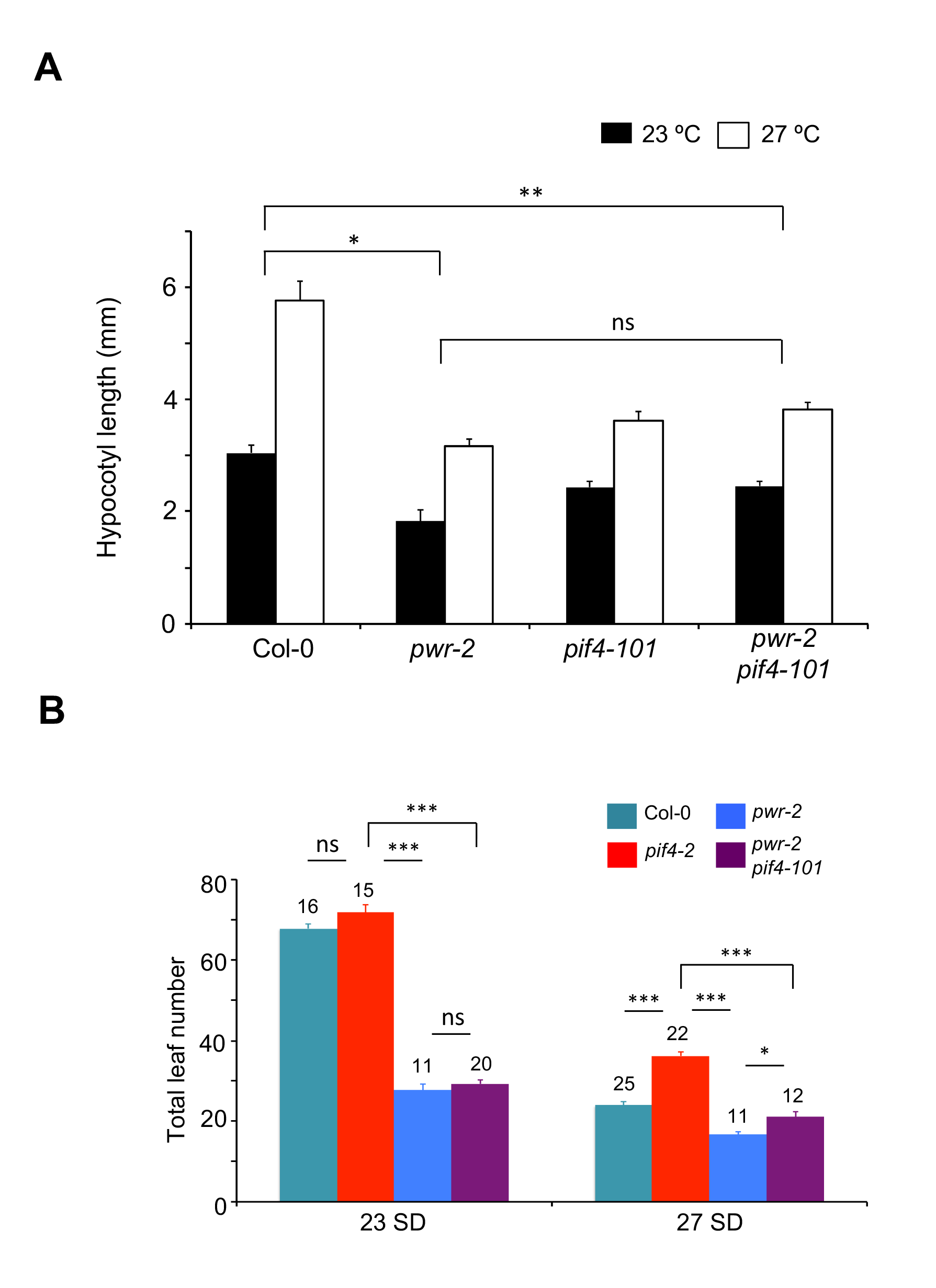

Supplement: S4 Fig — A) Hypocotyl lengths of various genotypes at 23°C and 27°C. p-values for the corresponding GxE interactions determined through ANOVA are shown. B) Flowering time of pif4-2 pwr-101 double mutants compared to single mutants at two different temperatures. Number of plants and the P-values determined through Student’s t-test are shown above bar graphs. Error bars indicate standard error. p- values: ***<0.0001, **<0.001, *<0.05, ns = not significant. (TIF) [file pgen.1007280.s004.tif]

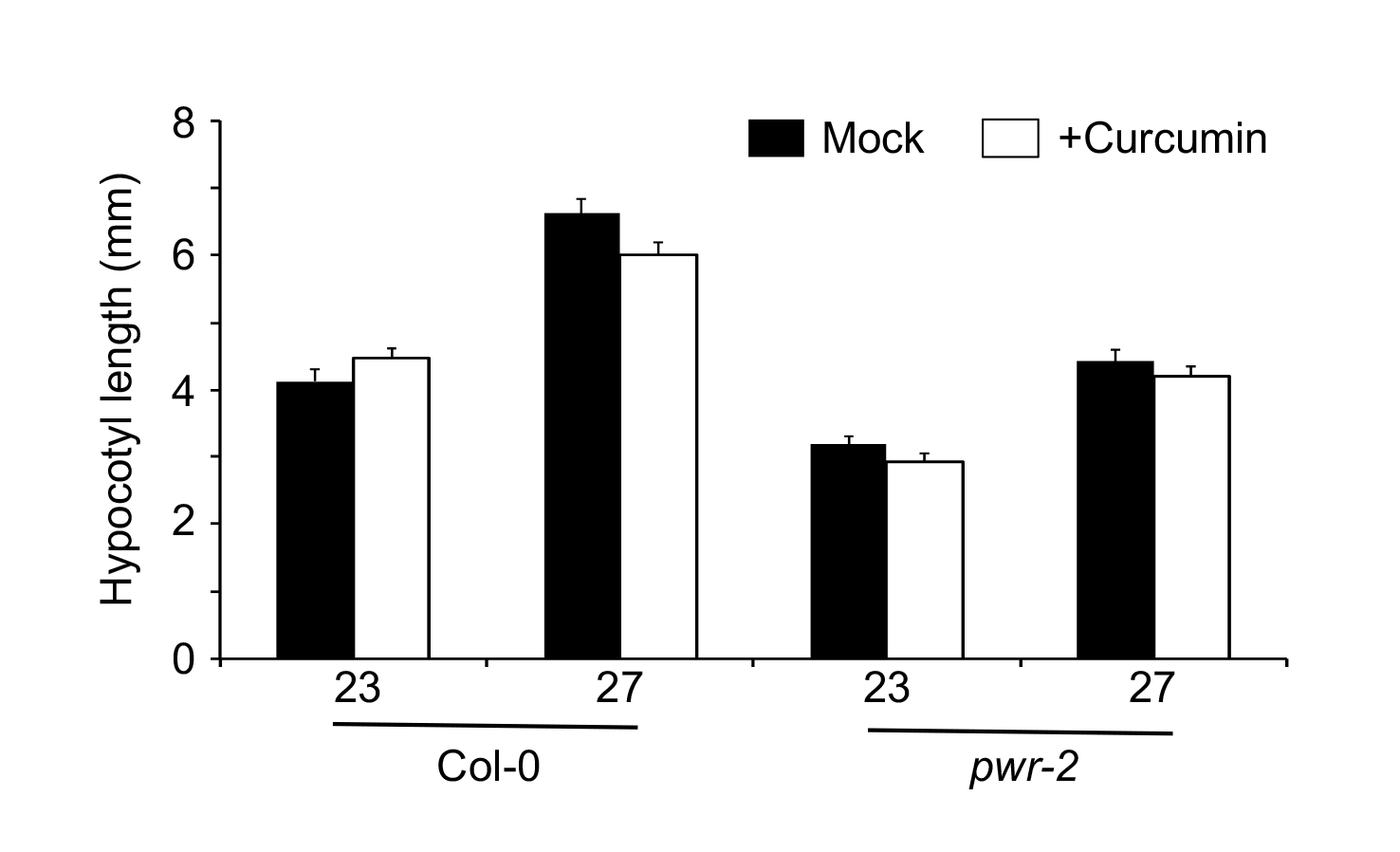

Supplement: S5 Fig — Hypocotyl elongation in Col-0 and pwr-2 mutants observed in plants grown in presence of 10uM of curcumin, an inhibitor or histone acetyl transferase compared to mock at 23°C and 27°C. (TIF) [file pgen.1007280.s005.tif]

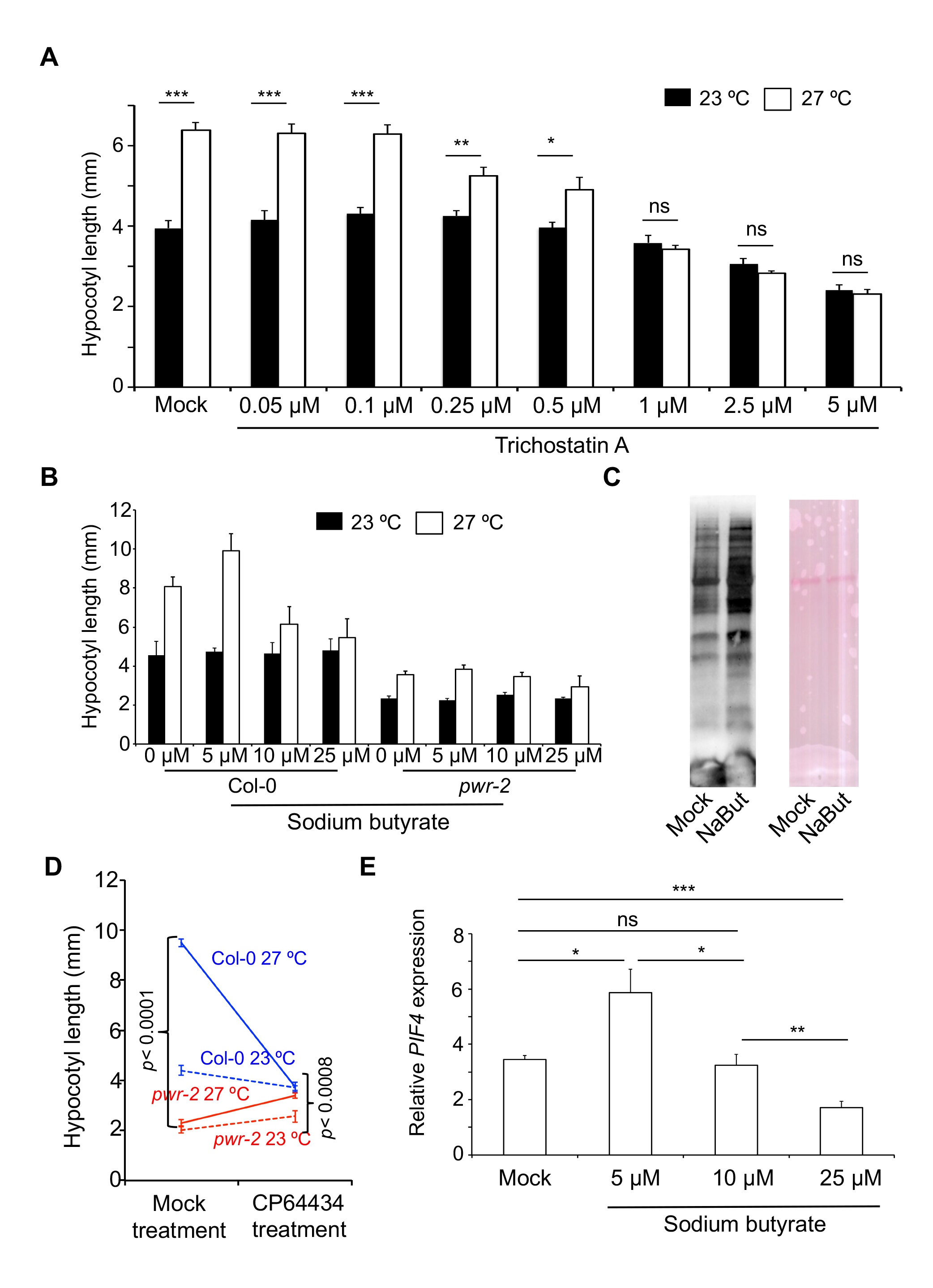

Supplement: S6 Fig — A & B) Dose-response effect of Trichostatin-A (A, N = 9 replicates with >20 plants), and Sodium Butyrate (B, N = 8), on Col-0 (A, B) and pwr-2 (B) at 23°C and 27°C. p-values shown are derived from one way ANOVA using the presence/absence of the drug as a factor. C) Western blots of crude plant extracts probed with anti-acetylated antibody from plants grown with or without Sodium Butyrate. Ponceau-S stained gel is shown as loading control. D) Reaction norms of hypocotyl lengths in Mock vs HDAC inhibitor treatment. The p-value for the drug x genotype interaction, determined through ANOVA is shown. E) Effect of inhibition of histone deacetylation on PIF4 expression in seedlings grown at 23°C or 27°C with or without Sodium Butyrate at different concentrations. p-values are determined by one way ANOVA with temperature as a factor. Error bars represent standard error. p- values: ***<0.0001, **<0.001, *<0.05, ns = not significant. (TIF) [file pgen.1007280.s006.tif]

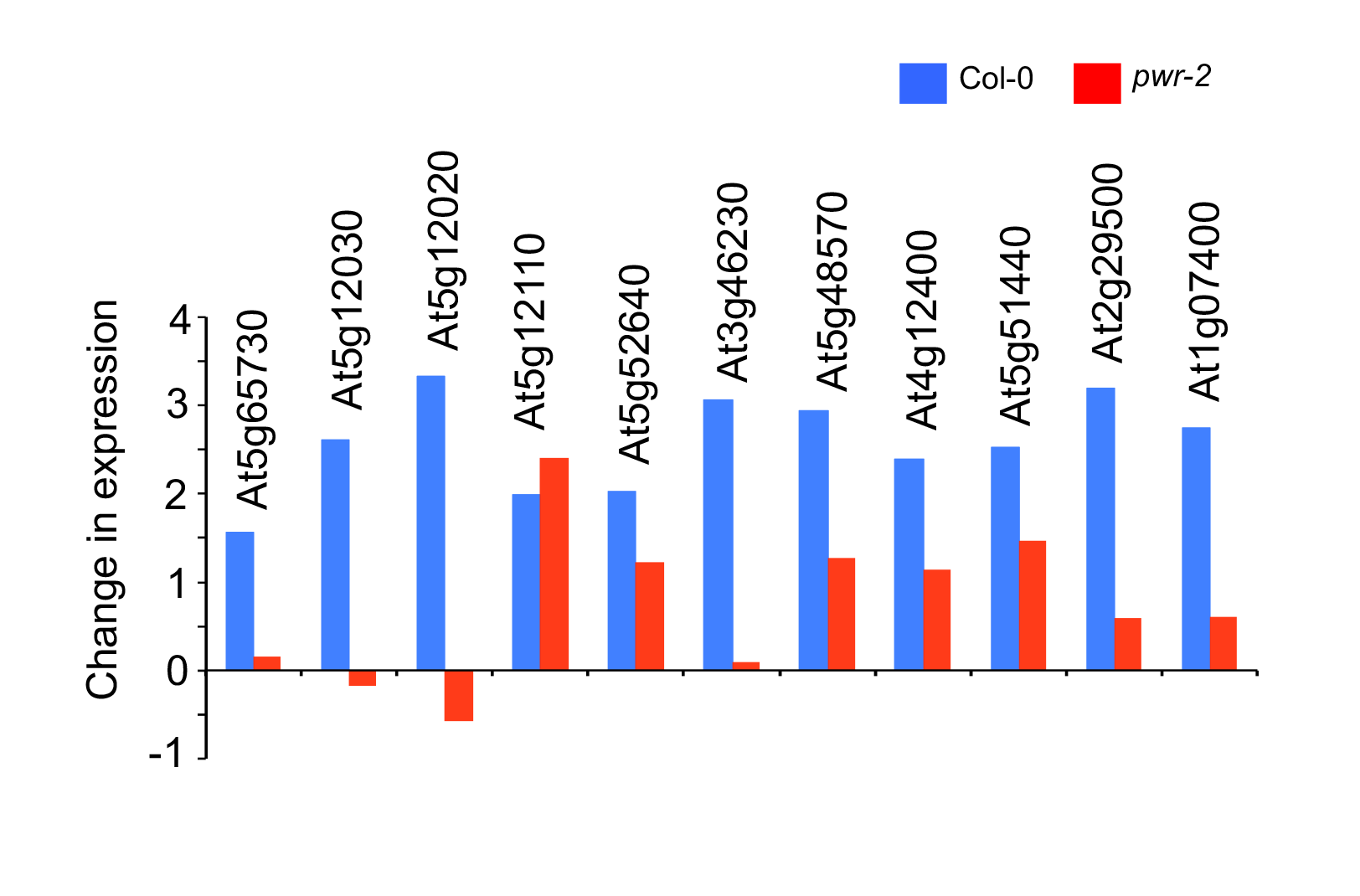

Supplement: S7 Fig — Comparison of the fold change in expression levels for the 11 genes that are up regulated at 27°C in Col-0 (blue) and their corresponding response in pwr-2 (red). (TIF) [file pgen.1007280.s007.tif]

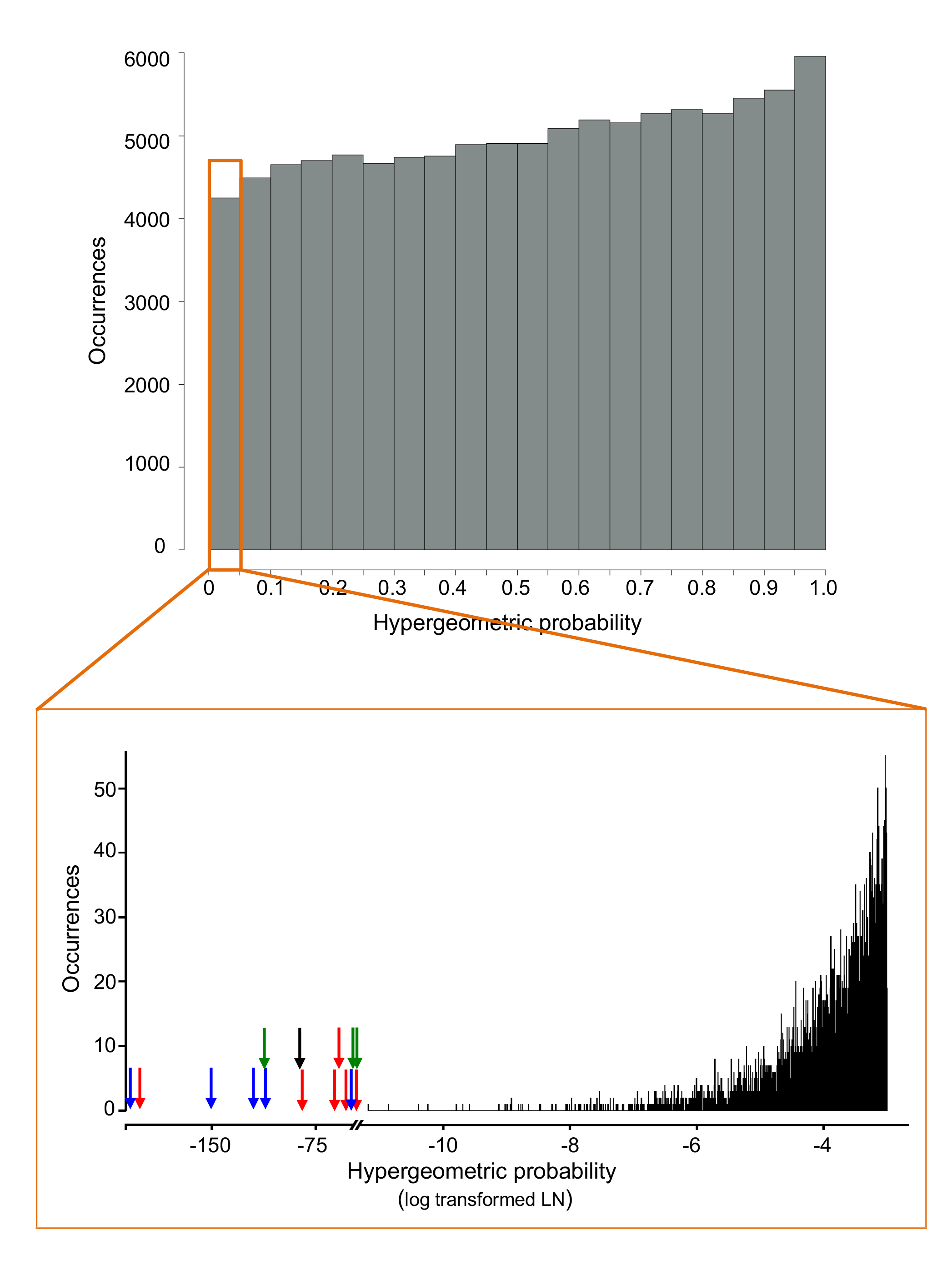

Supplement: S8 Fig — The top panel represents the frequency distribution of hypergeometric probabilities obtained through simulations. Random gene lists of 500 to 2000 genes were generated by sampling the Arabidopsis genome and the hypergeometric probability was estimated for the gene overlap. The entire analysis is repeated 100,000 times and the distribution of the probabilities is shown. To demonstrate the significance of the overlaps that are shown in this paper, the p-value distribution for those tests that yielded p<0.05 is shown in the second panel (expanded orange box). The p-values have been log-transformed to show the magnitude of the differences. Please note the differences in the scale for X-axis. The p-values for the gene overlaps for pwr-2 and hda9 from different labs is shown by green and black arrows respectively. The red-arrows depict the p-values for the gene overlap with DEGs in pwr-2 from our group and the blue arrows refer to the same for the overlap between the combined dataset for pwr-2 and other genes. Note all the p-values fall completely outside the distributions obtained through simulations. (TIF) [file pgen.1007280.s008.tif]

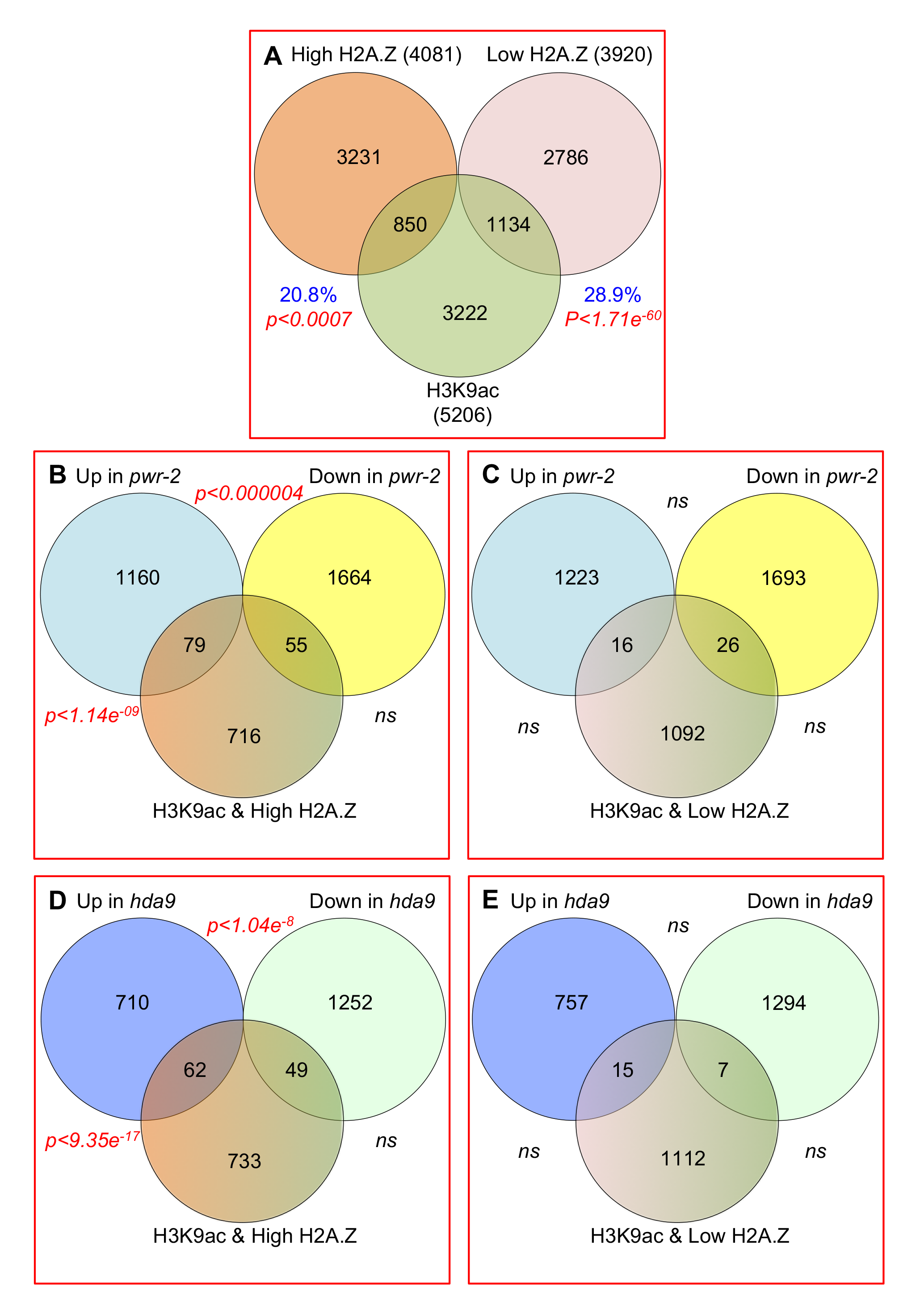

Supplement: S9 Fig — A) Overlap between genes with H3K9 acetylation and genes with either high or low H2A.Z in their gene bodies B & C) Overlap among genes that are up/down regulated in pwr-2 with H3K9acetylated genes with high (B) or low (C) H2A.Z. The DEGs data is the union of Tasset et al (current study) and Kim et al [25] study from seedlings. D & E) Overlap among genes that are up/down regulated in hda9 with H3K9acetylated genes with high (B) or low (C) H2A.Z. The DEGs data is from Kim et al [25]. The significant p-values shown in red represent hypergeometric probability for the overlap. ns = not significant. (TIF) [file pgen.1007280.s009.tif]

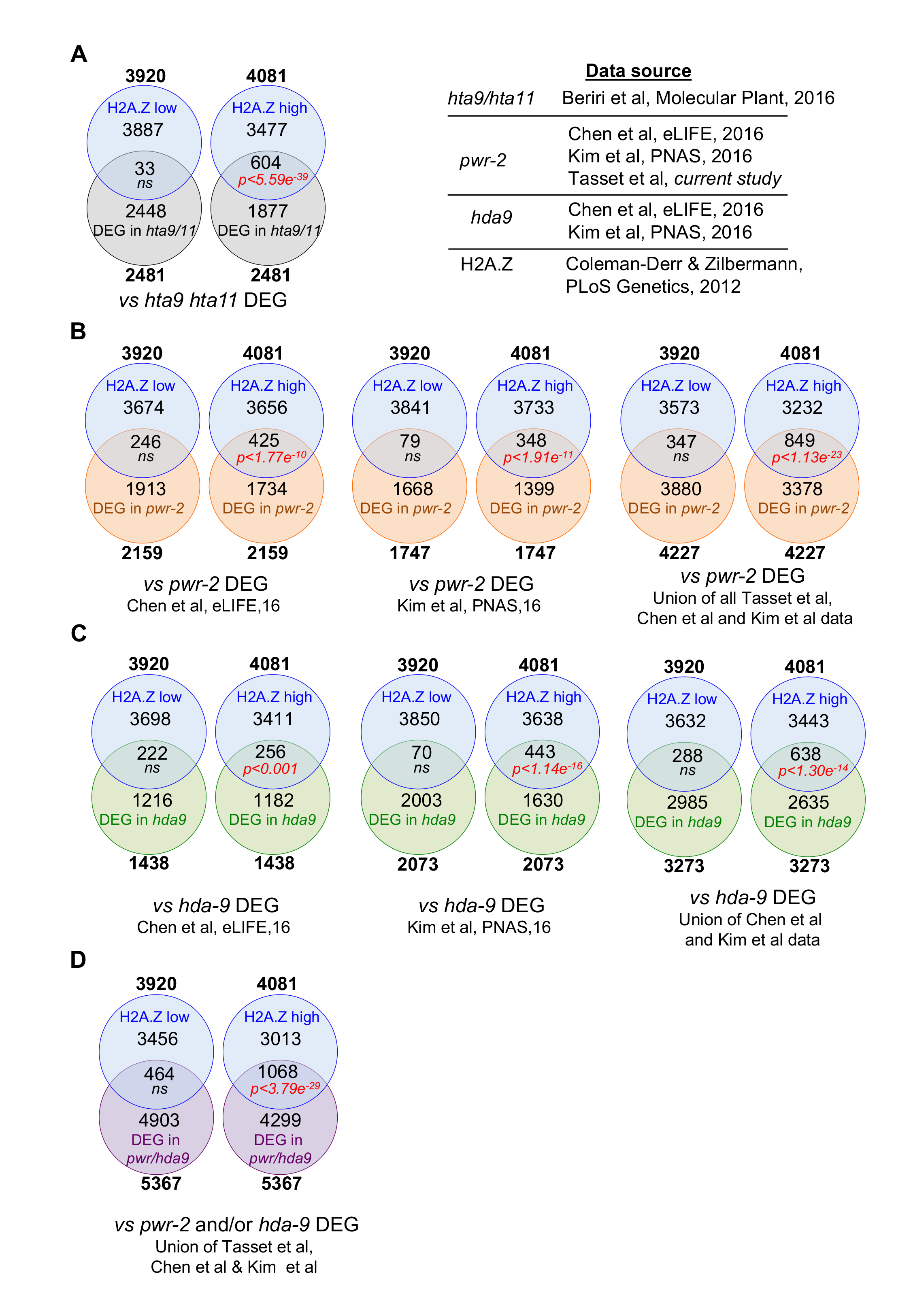

Supplement: S10 Fig — A) Overlap of the DEGs in hta9/hta11 double mutants with low-H2A.Z and high-H2A.Z genes. B) Overlap of DEGs in pwr-2 mutants from two different data sets with low-H2A.Z and high-H2A.Z genes are shown. C) Overlap of DEGs in hda9-1 mutants from two different data sets with low-H2A.Z and high H2A.Z genes are shown. D) Overlap of DEGs in pwr-2 and/or hda9 with low-H2A.Z and high-H2A.Z genes are shown. Total numbers of genes in respective gene lists are shown in bold. The data source is shown on top. p-values refer to hypergeometric probabilities and the significant p-values are shown in red. ns = not significant. (TIF) [file pgen.1007280.s010.tif]

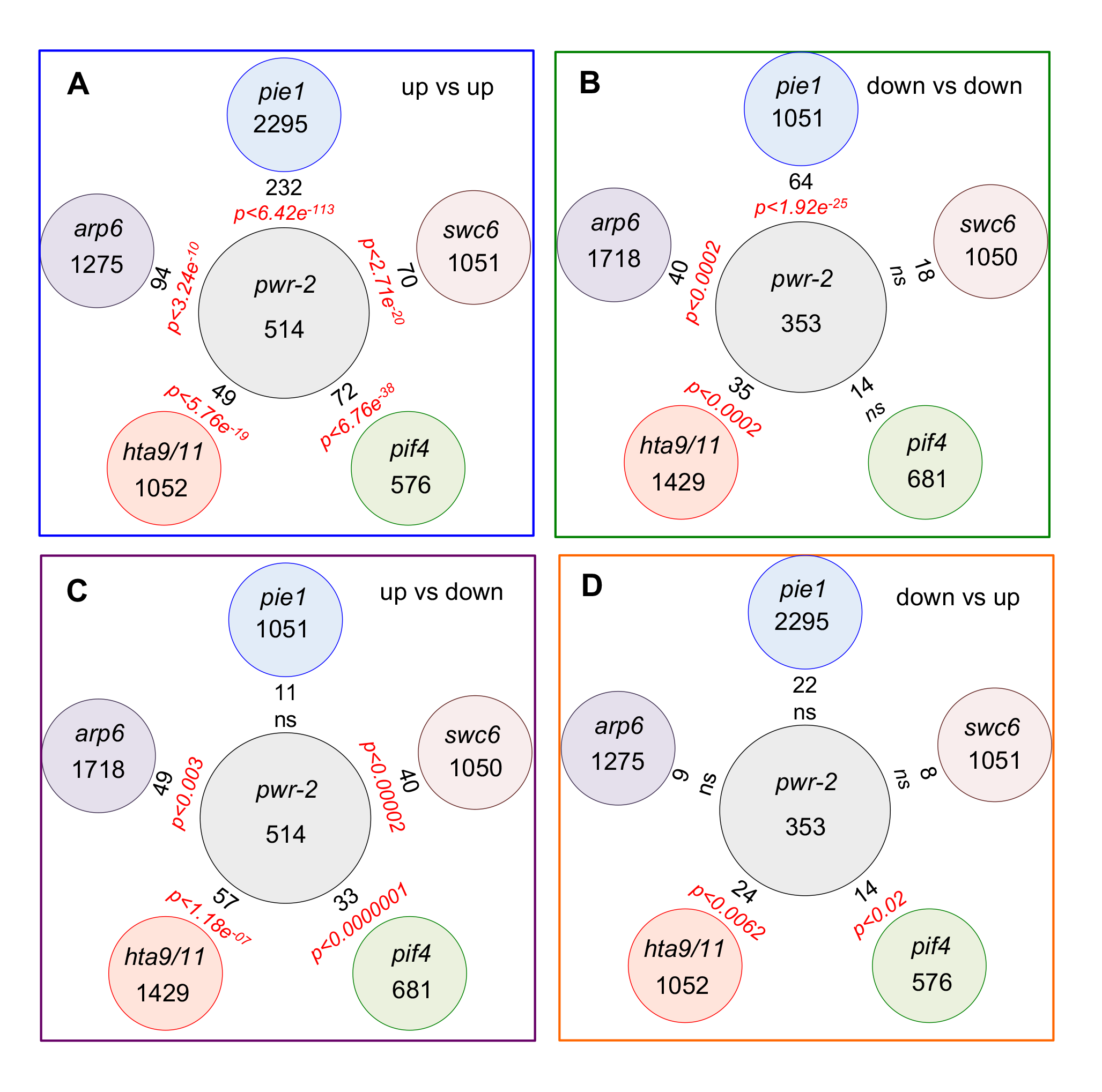

Supplement: S11 Fig — A-E) Overlap of DEGs in pwr-2 compared to Col-0 at 27°C with DEGs in pie1, swc6, pif4, hta9/hta11 and arp6. A) Overlap among DEGs. B) Overlap among genes that are up regulated in all genotypes. C) Overlap among genes that are down regulated in all genotypes. D) Overlap among genes that were up regulated in pwr-2, but down-regulated in other genotypes. E) Overlap among genes that were down-regulated in pwr-2, but up regulated in other genotypes. The total number of DEGs is shown in circles and the numbers in between represent the overlapping set of genes. The significant p-values shown in red represent hypergeometric probability for the overlap. ns = not significant. The transcriptome data is from[21, 34, 37]. (TIF) [file pgen.1007280.s011.tif]

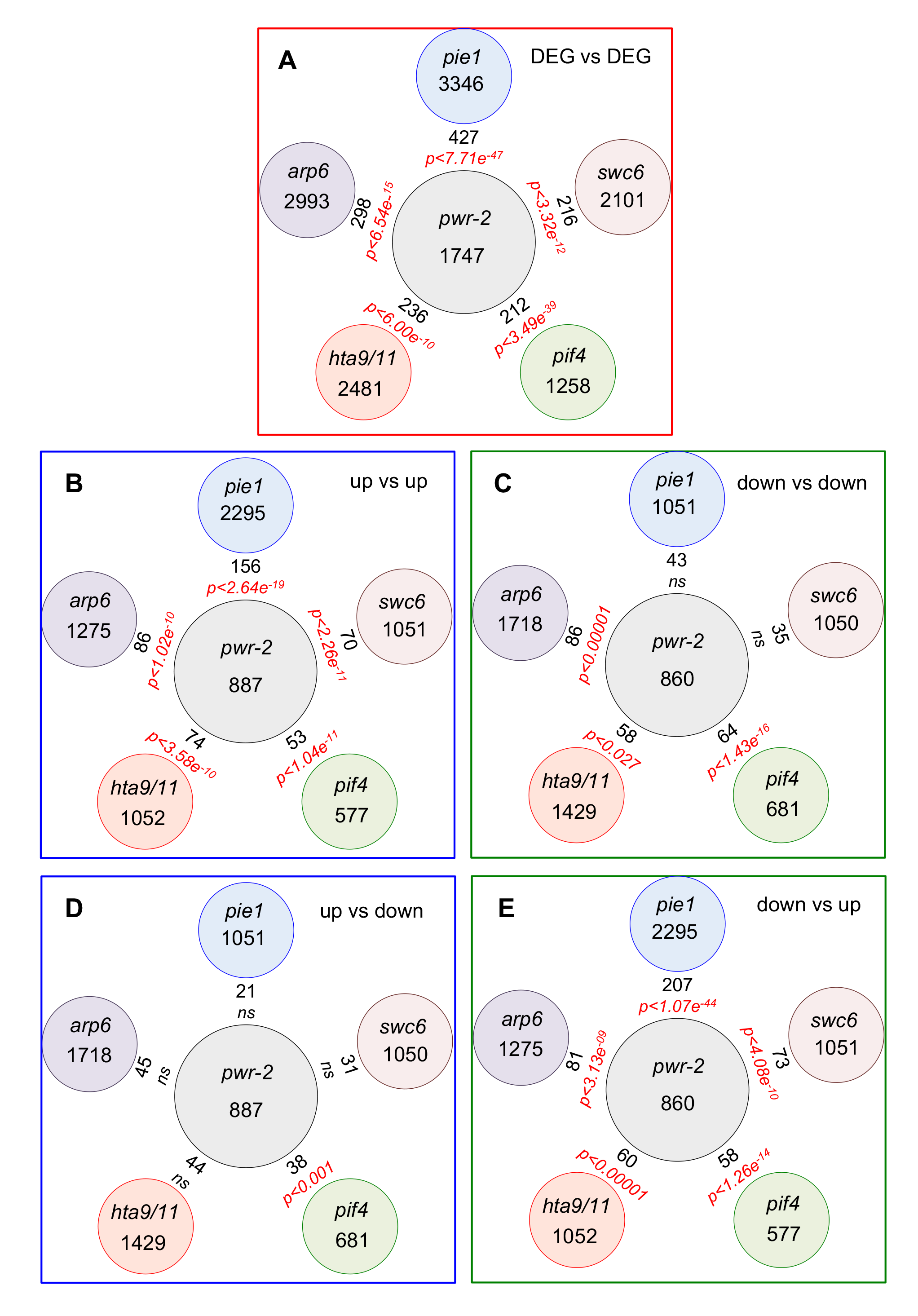

Supplement: S12 Fig — A-E) Overlap of DEGs in pwr-2 compared to Col-0 with DEGs in pie1, swc6, pif4, hta9/hta11 and arp6. A) Overlap among DEGs. B) Overlap among genes that are up regulated in all genotypes. C) Overlap among genes that are down regulated in all genotypes. D) Overlap among genes that were up regulated in pwr-2, but down regulated in other genotypes. E) Overlap among genes that were down regulated in pwr-2, but up regulated in other genotypes. The total number of DEGs is shown in circles and the numbers in between represent the overlapping set of genes. The significant p-values shown in red represent hypergeometric probability for the overlap. ns = not significant. The transcriptome data is from [21, 34, 37]. (TIF) [file pgen.1007280.s012.tif]

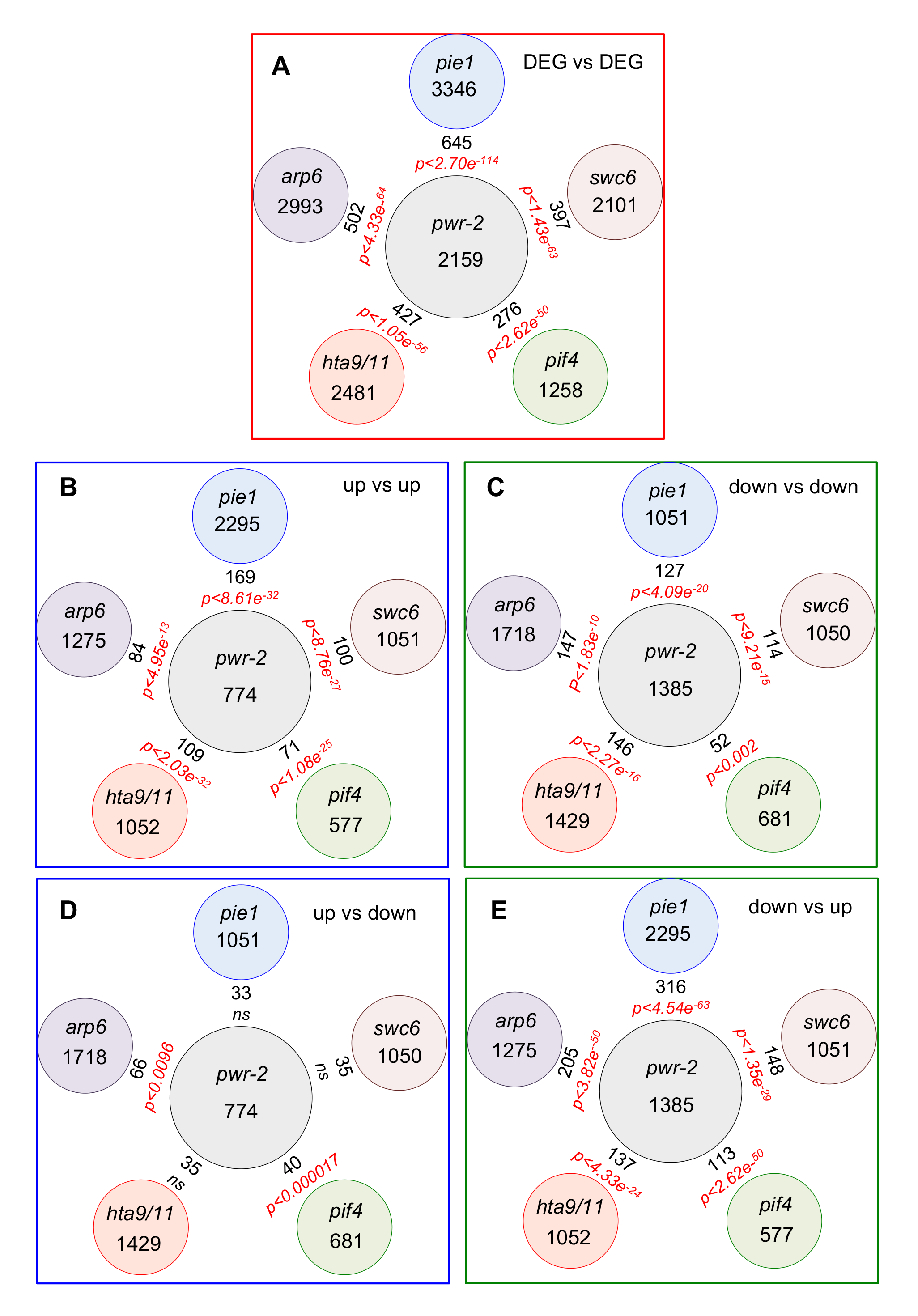

Supplement: S13 Fig — A-E) Overlap of DEGs in pwr-2 compared to Col-0 with DEGs in pie1, swc6, pif4, hta9/hta11 and arp6. A) Overlap among DEGs. B) Overlap among genes that are up regulated in all genotypes. C) Overlap among genes that are down regulated in all genotypes. D) Overlap among genes that were up regulated in pwr-2, but down regulated in other genotypes. E) Overlap among genes that were down regulated in pwr-2, but up regulated in other genotypes. The total number of DEGs is shown in circles and the numbers in between represent the overlapping set of genes. The significant p-values shown in red represent hypergeometric probability for the overlap. ns = not significant. The transcriptome data is from [21, 34, 37]. (TIF) [file pgen.1007280.s013.tif]

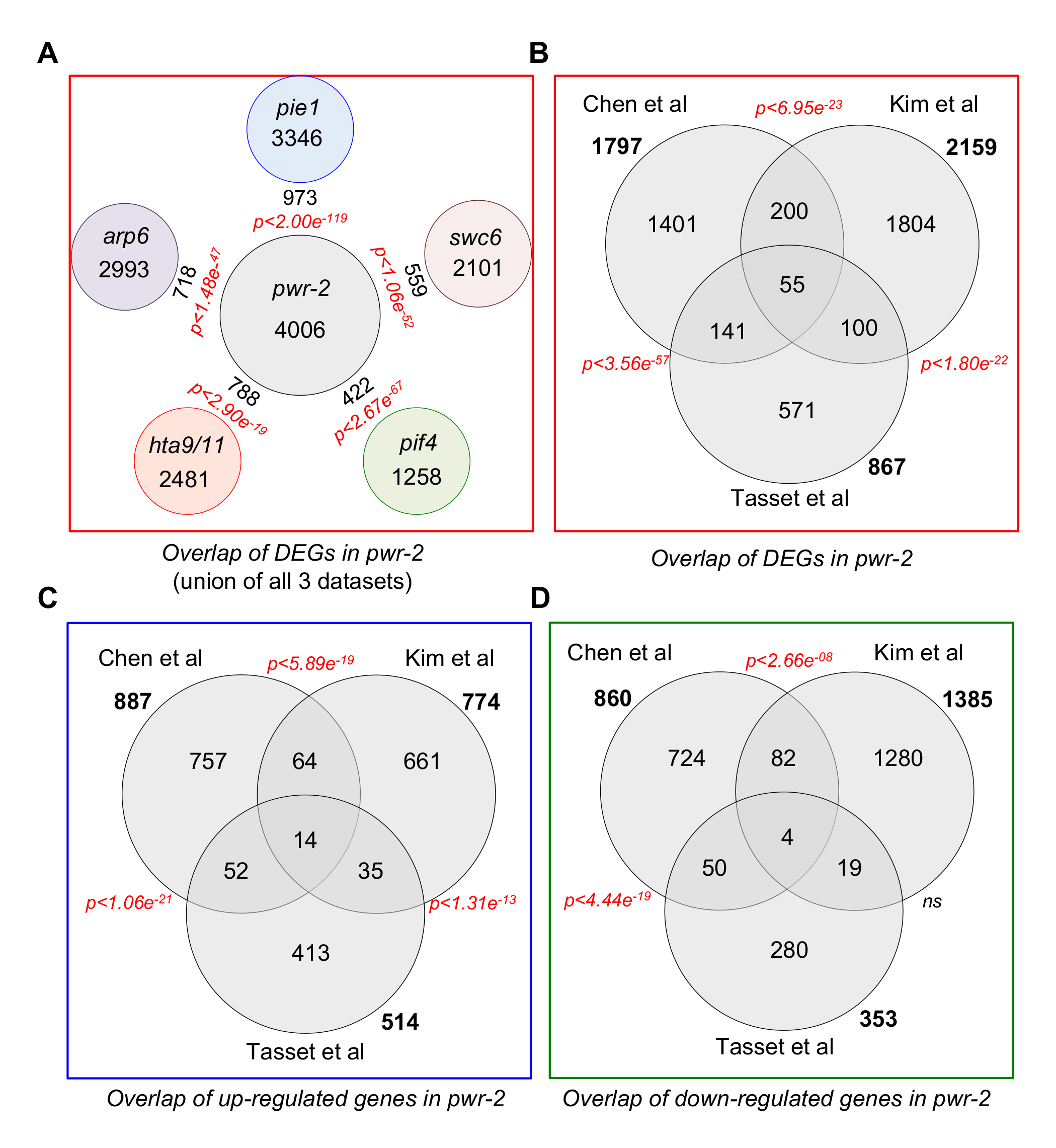

Supplement: S14 Fig — A) Overlap of DEGs in pwr compared to Col-0 compiled from all three datasets (excluding genes that did not change in the same direction in the datasets) with DEGs in pie1, swc6, pif4, hta9/hta11 and arp6. The total number of DEGs is shown in circles and the numbers in between represent the overlapping set of genes. The transcriptome data is from [21, 34, 37]. B) Overlap of the DEGs in pwr in the three different datasets. The transcriptome data is from [24, 25] The p-values are shown next to each of the overlaps. C) Overlap among up-regulated genes. D) Overlap among down regulated genes. The total number of DEGs is shown in circles and the numbers in between represent the overlapping set of genes (B-D). The significant p-values shown in red represent hypergeometric probability for the overlap. ns = not significant. (TIF) [file pgen.1007280.s014.tif]

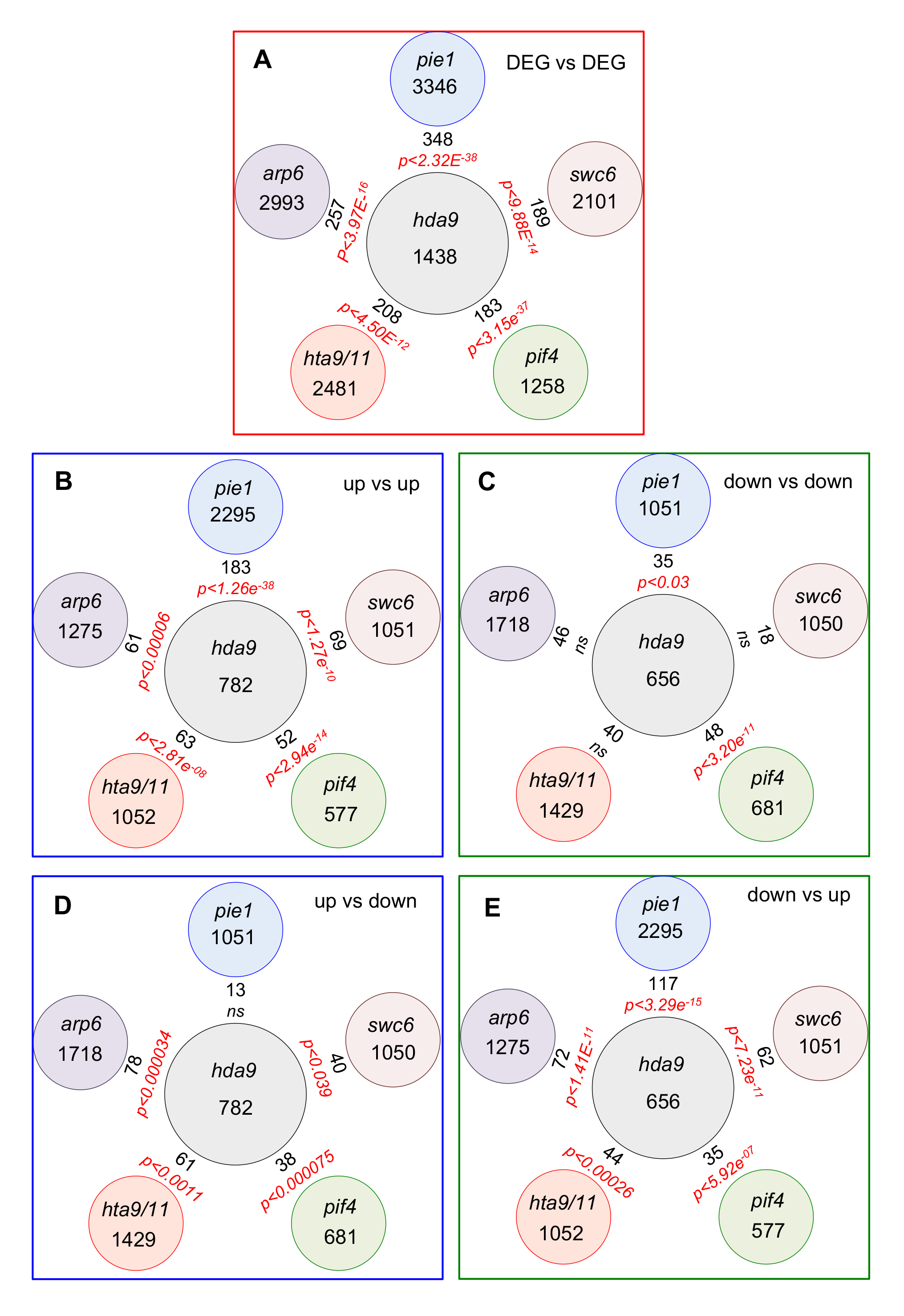

Supplement: S15 Fig — A-E) Overlap of DEGs in hda9 compared to Col-0 with DEGs in pie1, swc6, pif4, hta9/hta11 and arp6. A) Overlap among DEGs. B) Overlap among genes that are up regulated in all genotypes. C) Overlap among genes that are down regulated in all genotypes. D) Overlap among genes that were up regulated in hda9, but down regulated in other genotypes. E) Overlap among genes that were down regulated in hda9, but up regulated in other genotypes. The total number of DEGs is shown in circles and the numbers in between represent the overlapping set of genes. The significant p-values shown in red represent hypergeometric probability for the overlap. ns = not significant. The transcriptome data is from[21, 34, 37]. (TIF) [file pgen.1007280.s015.tif]

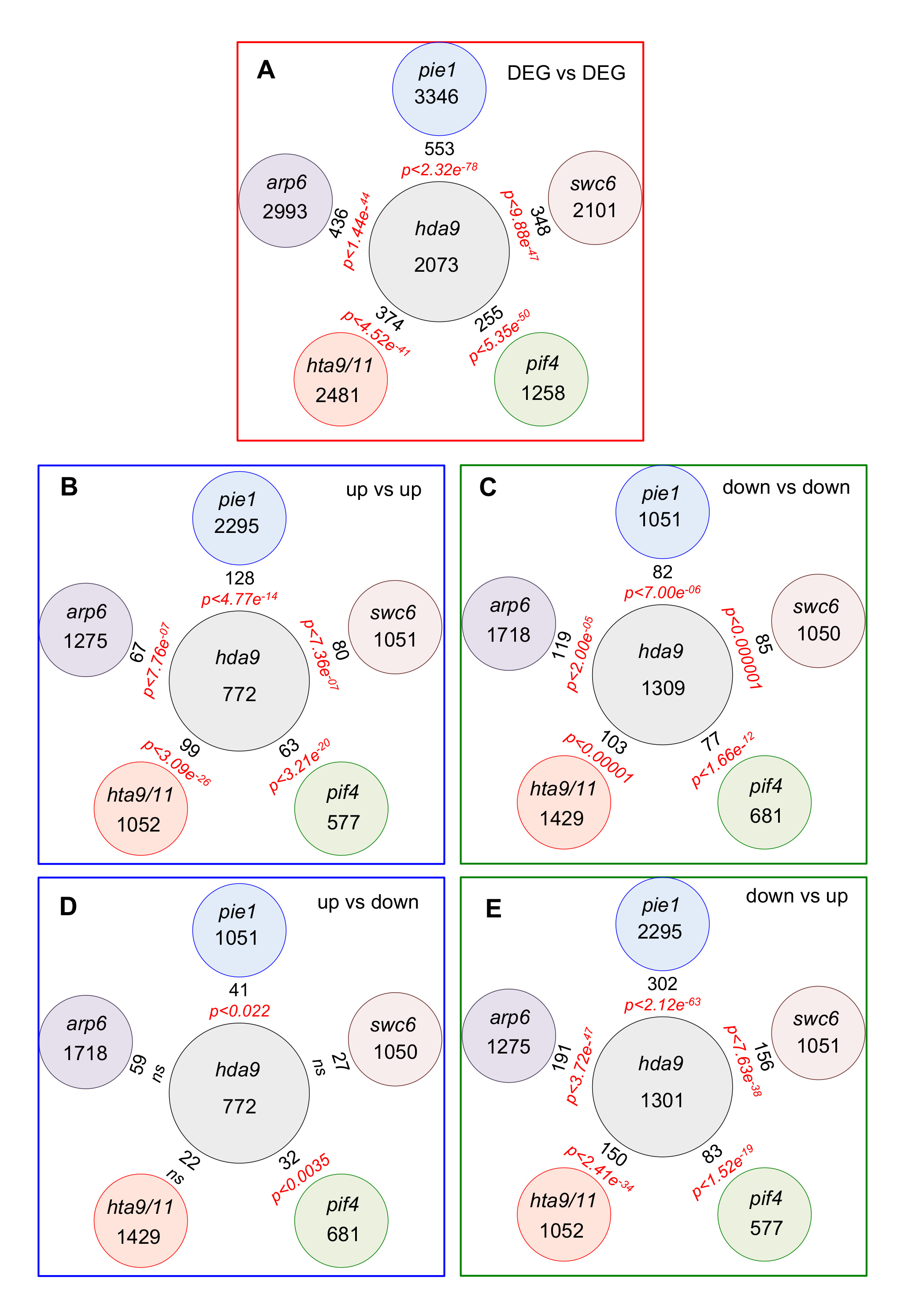

Supplement: S16 Fig — A-E) Overlap of DEGs in hda9 compared to Col-0 in the Kim et al data set with DEGs in pie1, swc6, pif4, hta9/hta11 and arp6. A) Overlap among DEGs. B) Overlap among genes that are up regulated in all genotypes. C) Overlap among genes that are down regulated in all genotypes. D) Overlap among genes that were up regulated in hda9, but down regulated in other genotypes. E) Overlap among genes that were down regulated in hda9, but up regulated in other genotypes. The total number of DEGs is shown in circles and the numbers in between represent the overlapping set of genes. The significant p-values shown in red represent hypergeometric probability for the overlap. ns = not significant. The transcriptome data is from[21, 24, 25, 34, 37]. (TIF) [file pgen.1007280.s016.tif]

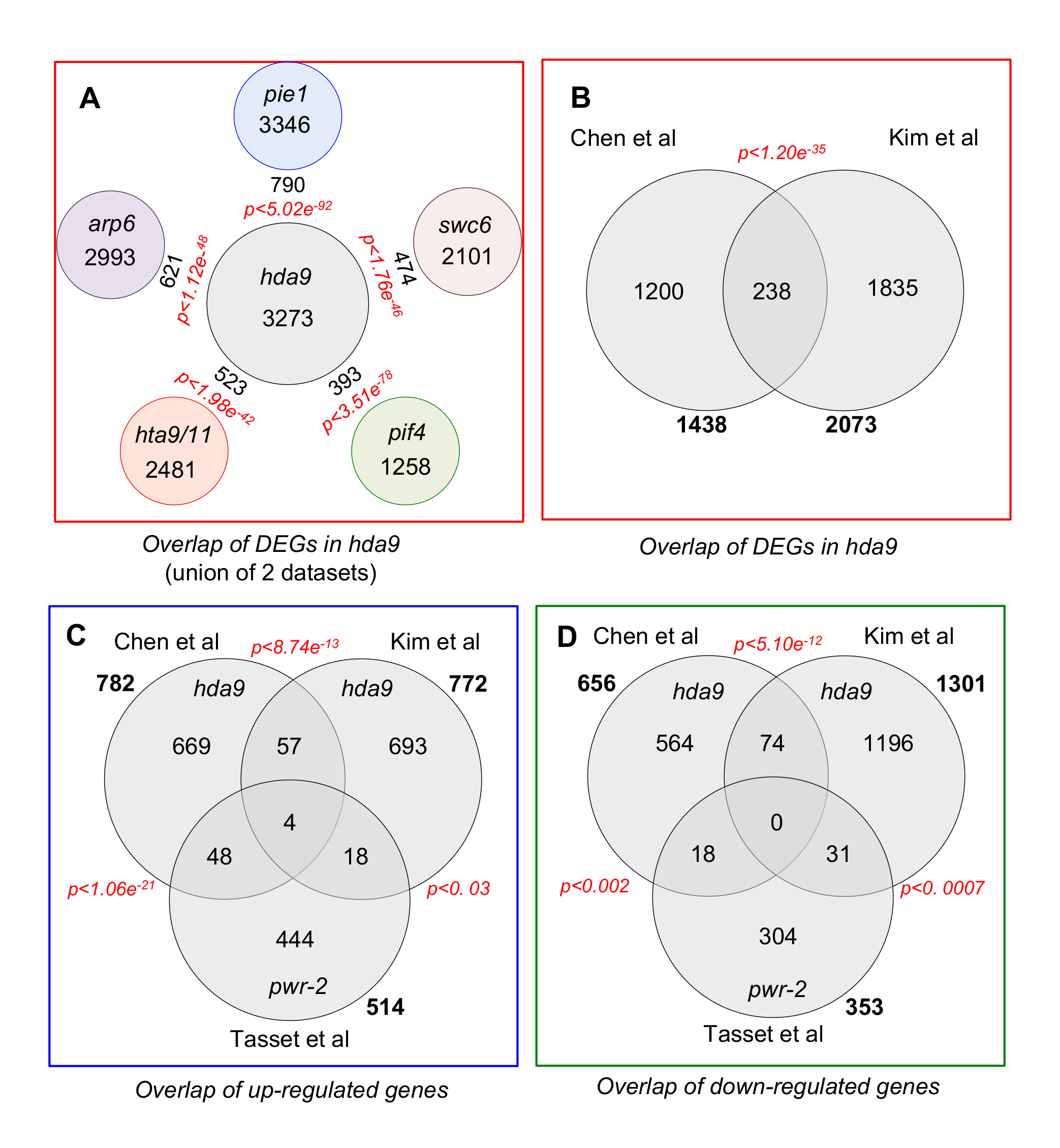

Supplement: S17 Fig — A) Overlap of DEGs in hda9 compared to Col-0 compiled from two datasets (excluding genes that did not change in the same direction in the datasets) with DEGs in pie1, swc6, pif4, hta9/hta11 and arp6. The total number of DEGs is shown in circles and the numbers in between represent the overlapping set of genes. The transcriptome data is from [21, 24, 25, 34, 37]. B) Overlap of the DEGs in pwr in the three different datasets. The p-values are shown next to each of the overlaps. C) Overlap among up-regulated genes. D) Overlap among down regulated genes. The significant p-values shown in red represent hypergeometric probability for the overlap. ns = not significant. (TIF) [file pgen.1007280.s017.tif]

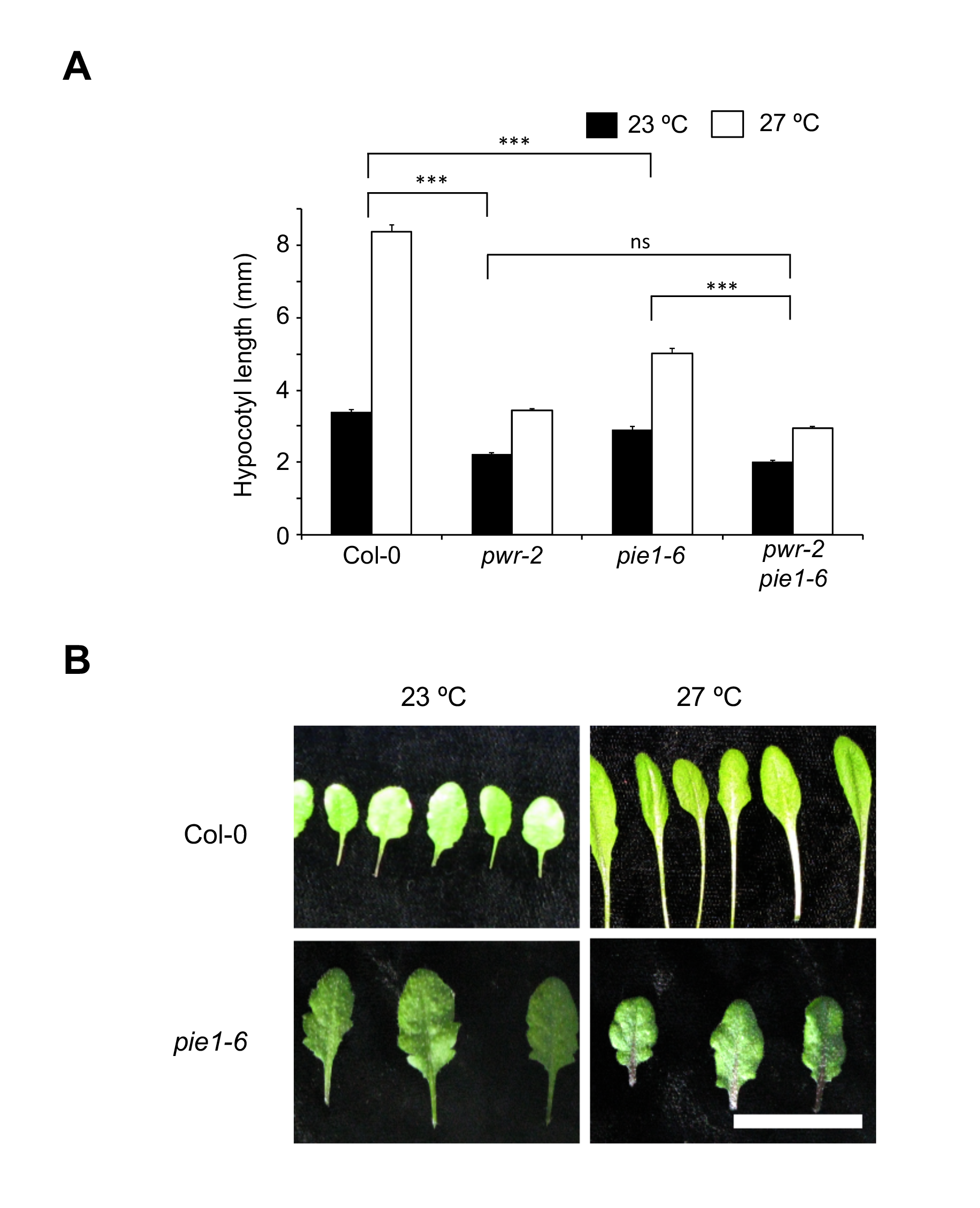

Supplement: S18 Fig — A) Hypocotyl lengths of various genotypes at 23°C and 27°C. P-values for the corresponding GxE interactions determined through ANOVA are shown. The Col-0 and pwr-2 data is the same as shown in Fig 2A. B) Comparison of the Col-0 and pie1 mutant leaves grown at 23°C and 27°C. Scale bar: 1cm. Error bars indicate standard error. p- values: ***<0.0001, **<0.001, *<0.05. (TIF) [file pgen.1007280.s018.tif]
